# Base editing enables duplex point mutagenesis in *Clostridium autoethanogenum* at the price of numerous off-target mutations

**DOI:** 10.3389/fbioe.2023.1211197

**Published:** 2023-07-10

**Authors:** François M. Seys, Christopher M. Humphreys, Claudio Tomi-Andrino, Qi Li, Thomas Millat, Sheng Yang, Nigel P. Minton

**Affiliations:** ^1^ Clostridia Research Group, BBSRC/EPSRC Synthetic Biology Research Centre (SBRC), School of Life Sciences, Biodiscovery Institute, University of Nottingham, Nottingham, United Kingdom; ^2^ Centre for Analytical Bioscience, Advanced Materials and Healthcare Technologies Division, School of Pharmacy, University of Nottingham, Nottingham, United Kingdom; ^3^ Nottingham BBSRC/EPSRC Synthetic Biology Research Centre (SBRC), School of Mathematical Sciences, University of Nottingham, Nottingham, United Kingdom; ^4^ College of Life Sciences, Sichuan Normal University, Chengdu, China; ^5^ Key Laboratory of Synthetic Biology, CAS Center for Excellence in Molecular Plant Sciences, Chinese Academy of Sciences, Shanghai, China

**Keywords:** base editor, target-AID, *Clostridium*, multiplex mutagenesis, Cas9, off-target mutagenesis, alternative PAM, tRNA

## Abstract

Base editors are recent multiplex gene editing tools derived from the Cas9 nuclease of *Streptomyces pyogenes*. They can target and modify a single nucleotide in the genome without inducing double-strand breaks (DSB) of the DNA helix. As such, they hold great potential for the engineering of microbes that lack effective DSB repair pathways such as homologous recombination (HR) or non-homologous end-joining (NHEJ). However, few applications of base editors have been reported in prokaryotes to date, and their advantages and drawbacks have not been systematically reported. Here, we used the base editors Target-AID and Target-AID-NG to introduce nonsense mutations into four different coding sequences of the industrially relevant Gram-positive bacterium *Clostridium autoethanogenum*. While up to two loci could be edited simultaneously using a variety of multiplexing strategies, most colonies exhibited mixed genotypes and most available protospacers led to undesired mutations within the targeted editing window. Additionally, fifteen off-target mutations were detected by sequencing the genome of the resulting strain, among them seven single-nucleotide polymorphisms (SNP) in or near loci bearing some similarity with the targeted protospacers, one 15 nt duplication, and one 12 kb deletion which removed uracil DNA glycosylase (UDG), a key DNA repair enzyme thought to be an obstacle to base editing mutagenesis. A strategy to process prokaryotic single-guide RNA arrays by exploiting tRNA maturation mechanisms is also illustrated.

## 1 Introduction

Base editors are recent gene editing tools derived from the Cas9 nuclease of *Streptomyces* pyogenes (Komor et al., 2016; Nishida et al., 2016). They can target and modify a single nucleotide in the genome without inducing double-strand breaks (DSB) of the DNA helix. This can be exploited to change a single amino acid for *in vivo* protein engineering, or, more commonly, to disrupt protein expression with nonsense mutations. Consequently, base editors are promising tools for the engineering of microbes that lack effective DSB repair pathways such as homologous recombination (HR) or non-homologous end-joining (NHEJ) (Wang et al., 2018; Luo et al., 2020). Additionally, base editors are simple to customize to a new target gene, requiring only to swap their 20 nt protospacer. This makes them readily compatible with high-throughput automated workflows (Wang et al., 2018). These combined features make them ideal candidates for multiplex genome editing tools, allowing the engineering of several loci in a single step and thereby significantly reducing the duration, cost and effort involved in mutagenesis procedures (Wang et al., 2020). In view of their advantages, in the current study the potential of base editors as multiplex genome editing tools was tested in *Clostridium* autoethanogenum. This Gram-positive acetogen is the process chassis used in the large-scale, commercial manufacture of ethanol from industrial off-gas (Fackler et al., 2021; Liew et al., 2022), characterised as slow-growing and challenging to engineer (Bourgade et al., 2021).

**FIGURE 1:**
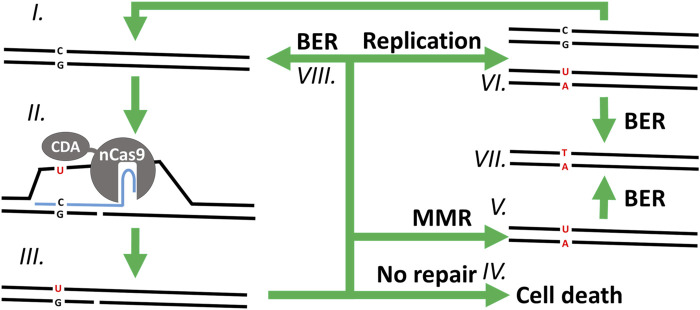
Overview of the proposed mutagenesis mechanism of CBEs. In a clockwise order starting from (I.) with the WT chromosomic DNA. (II.) The CBE-sgRNA duplex unwinds the DNA double helix around the protospacer, creating a R-loop, nicking the non-edited strand, and exposing a cytosine (C) to the deaminase activity of CDA on the edited strand—which (III.) changes it into uracil (U). *(IV.)* Without repair of the nick on the non-edited strand, the cell is unable to replicate its DNA and dies. *(V.)* If the DNA helix undergoes MMR, the nicked non-edited strand is repaired to match the edited strand, replacing the guanine (G) of the non-edited strand with an adenine (A). (VI.) Alternatively, the nick can be ligated, and the DNA replicated semi-conservatively into two double helixes: one mutated and the other one WT. (VII.) In the last step, the uracil of the edited strand is finally removed and replaced by a thymine (T) through DNA replication (not shown) or BER. Finally, immediate ligation of the nicked strand and subsequent repair of the edited strand through (VIII.) BER (or (VI.) DNA replication) would result in a stable WT chromosome which would be exposed to another cycle of mutagenesis for as long as the base editor and its sgRNA cassette are being expressed.

Several base editors have been developed to date, enabling C-to-T (cytosine base editors, CBE) or A-to-G (adenine base editors, ABE) (Gaudelli et al., 2017) targeted point mutations within various editing windows around the protospacer-adjacent motifs (PAMs) of their respective CRISPR-associated (Cas) nucleases (Banno et al., 2018; Eid et al., 2018; Jiang et al., 2018; Li et al., 2018; Marx, 2018; Molla & Yang, 2019; Chatterjee et al., 2020). Target-AID, a CBE, exploits the targeting capabilities of single guide RNAs (sgRNAs) and the activity of a cytidine deaminase (CDA), to deaminate a cytosine on a single strand of the DNA helix (Figure 1) in between the positions −20 and −16 from a PAM with the sequence NGG (Nishida et al., 2016). Then, the mismatch repair pathway (MMR) is hijacked by nicking the unedited strand with Cas9^D10A^ nickase (nCas9 ^D10A^), which is thought to trick the MMR pathway into using the edited strand as template to repair the unedited strand (Modrich, 2006; Li, 2008; Spampinato et al., 2009; Fukui, 2010; Williams & Kunkel, 2014). However, the base excision repair pathway (BER), initiated by the specialized enzyme uracil DNA glycosylase (UDG), can sometimes remove the deaminated cytosine before the MMR pathway has had a chance mutate the unedited strand. For this reason, many CBEs are fused to another enzyme called uracil glycosylase inhibitor (UGI) to protect the targeted locus from UDG and the BER pathway during the early stages of mutagenesis (Zhigang et al., 1991; Komor et al., 2016; Nishida et al., 2016; Wang et al., 2017).

Unfortunately, UGI has also been associated with extra toxicity and off-target mutagenesis in prokaryotes, even after the addition of a GLVA protein degradation tag (Banno et al., 2018). Consequently, we opted to avoid using a UGI fusion in the initial stages of our study. It has also been reported that altering the size of the spacer region of the sgRNA could change the base editing profile of Target-AID in *Escherichia coli*: instead of preferentially mutating the base in position −18 from the PAM with a standard 20 nt spacer, a 18 nt spacer would preferentially edit the base in position −17, and a 22 nt spacer would preferably edit the base in position −19 (Banno et al., 2018). We opted to test if this flexibility could also be exploited in *Clostridium autoethanogenum*.

To take full advantage of Target-AID’s simplicity, several multiplexing strategies (Adiego-Pérez et al., 2019) that could target several protospacers at once with a single plasmid were also tested. Among more traditional strategies such as using multiple sgRNA transcriptional units (msgRNA) and reproducing the native SpCas9 CRISPR array (mCRISPR), the exploitation of a polycistronic array of sgRNAs and prokaryotic tRNAs fusions (mtRNA) was also explored with the expectation that individual sgRNAs would be released as a consequence of tRNA maturation. This is a common strategy in eukaryotic systems (Xie et al., 2015; Dong et al., 2017; Zhang et al., 2019), but, to the best of our knowledge, it has not yet been used in prokaryotes except for a single recent report in a nonmainstream journal (Lu et al., 2022).

Finally, and perhaps most importantly, the mutant strains were validated by whole-genome sequencing to look for potential off-target mutations.

## 2 Materials and methods

### 2.1 Strains and media

Vector assembly and cloning was conducted in *E. coli* strain DH5α, cultivated in Luria-Bertani (LB) broth. *E. coli* strain sExpress (Woods et al., 2019) was used as the conjugal DNA donor strain to transfer plasmids into *C. autoethanogenum* strain DSM10061. *C. autoethanogenum* was recovered from cryostocks and cultivated in pre-reduced yeast tryptone fructose (YTF) medium in an anaerobic cabinet (Don Whitley Scientific Ltd., Bingley, United Kingdom) at 37°C (Humphreys et al., 2015). Antibiotics and other additives to LB and YTF are summarized in Table 1.

**TABLE 1:** Antibiotics and additives used in this study.

Additive	Abbreviation	Working concentration (μg/mL)	Purpose
Chloramphenicol	Cm	12.5	Maintains pMTL83151-derived plasmids in *E. coli*
Kanamycin	Kan	50	Maintains the conjugative R702 plasmid in *E. coli*
D-cycloserine	D-cyc	250	Counter-selects *E. coli* sExpress during transconjugants selection
Thiamphenicol	Tm	7.5	Maintains pMTL83151-derived plasmids in *C. autoethanogenum*
Theophylline	Th	900	Induces Target-AID in *C. autoethanogenum*
5-Fluoroorotic acid	5-FOA	1,000	Selects for *ΔpyrE* genotype

The mutant *C. autoethanogenum* strains generated over the course of this study are summarised in Table 2.

**TABLE 2:** Mutant strains generated in this study.

Strain	Background	Genotype	Purpose	Engineered with plasmid
*cFS02*	*C. autoethanogenum*	*pyrE* (Q130X)	Proof of concept of initial Target-AID construct and knock-out strategy	vFS36
*cFS04*	*C. autoethanogenum*	*CLAU_532*(Q243X), *CLAU_534*(A242V)	Proof of concept of multiplex Target-AID	vFS50
*cFS05*	*C. autoethanogenum*	*CLAU_532*(Q243X), *CLAU_534*(A242V), *CLAU_1794*(S251L, W278X)	Proof of concept of multiplex Target-AID-NG	vFS50, vFS74
*cFS15*	*C. autoethanogenum*	*CLAU_532*(Q243X), *CLAU_534*(A242X)	Proof of concept of multiplex Target-AID	vFS50

### 2.2 Cloning and assembly

*In silico* design of constructs was achieved with A plasmid Editor (ApE) (RRID:SCR_014266) (Davis & Jorgensen, 2022). Unless otherwise specified, all kits, enzymes and buffers were purchased from New England Biolabs Ltd. (NEB, Hitchin, United Kingdom) and used following the manufacturer’s instructions. DNA oligos were synthesised by Integrated DNA Technologies, Inc. (Coralville, United States) and were designed to have an annealing region with a melting temperature of 65°C using NEB Tm calculator (RRID:SCR_017969, tmcalculator.neb.com) and no secondary structure with a melting temperature higher than 57°C (modelled with Mfold, RRID:SCR_008543 (Zuker, 2003)). All oligos and vectors used in this study are summarized in Supplementary Tables S1, S2. Amplification of all parts was achieved by polymerase chain reactions (PCR) with Q5^®^ high-fidelity polymerase 2x master mix and an annealing temperature of 60°C. All parts were assembled with NEBuilder^®^ HiFi assembly, designed to share between 25 nt and 40 nt of homologous overlap, and purified using the NEB Monarch^®^ DNA gel purification kit. All vectors were transformed into chemically competent *E. coli* DH5α following NEB’s protocol. *E. coli* colony PCR was done with DreamTaq Green PCR Master Mix (2X) from Thermofisher Scientific (Waltham, United States) after resuspending each colony into 40 μL of sterile ddH2O and using 1 μL as DNA template. After gel electrophoresis of the PCR products to screen for amplicons of the right size, these same colony aliquots were used to inoculate overnight cultures before proceeding to cryopreservation in Microbank^®^ from Pro-lab diagnostics (Richmond Hill, Canada) and plasmid extraction with NEB Monarch^®^ plasmid miniprep kit. Plasmids were then subjected to Sanger sequencing by Eurofins Genomics (Ebersberg, Germany). A detailed description of all cloning steps for each vector is available in the Supplementary Material, alongside key sequences highlighted in the text in Supplementary Tables S3, S4, S5. The full sequence of each vector is available upon request and at www.plasmidvectors.com (RRID: SCR_023475) where plasmids may be sourced.

#### 2.2.1 Codon optimization

The sequence coding for UGI-GLVA and the activation-induced cytidine deaminase 1 from *Petromyzon marinus* (PmCDA1, or AID) with its long protein linker (Nishida et al., 2016; Banno et al., 2018) were codon optimized by Genscript (Piscataway, United States) to match the codon usage of *C. autoethanogenum*. The codon utilization table of *C. autoethanogenum* was obtained by extracting all the coding sequences (CDS) from its published genome (GenBank: CP012395.1) (Humphreys et al., 2015) and processing them with the CUSP algorithm (Rice et al., 2000).

#### 2.2.2 Design of a *Clostridium* Target-AID plasmid

The Target-AID CDS was generated by replacing the STOP codon of the Cas9^D10A^ nickase (nCas9) CDS with the CDS of the codon-optimized AID and its long protein linker (Nishida et al., 2016). Target-AID was then cloned inside the pMTL83151 backbone of our RiboCas system (Cañadas et al., 2019). Consequently, expression of Target-AID was subordinated to the tight control of a theophylline-inducible riboswitch, and expression of the custom sgRNA was driven by the strong P*araE* constitutive promoter (Huang et al., 2016).

#### 2.2.3 Protospacer design and modelling of total protospacers and genomic coverage

Potential Target-AID protospacer targets for each gene were initially identified with Benchling (RRID:SCR_013955) CRISPR design tool, which lists all the 20 nt sequences directly upstream of an NGG PAM in a given DNA sequence. This list was then pasted onto a custom Microsoft Office Excel™ spreadsheet, which flagged the protospacers with the appropriate cytosines in positions −19 to −16 from the PAM that could lead to a TAA, TAG or TGA codon if turned into a thymine. Target-AID-NG protospacers were pulled from our whole-genome analysis of potential Target-AID targets for various PAMs in *C. autoethanogenum* using MATLAB™ (RRID:SCR_001622). For future work on individual genes, we recommend the use of BE-designer (RRID:SCR_023389, rgenome.net/be-designer/), an online tool with a user-friendly interface.

#### 2.2.4 Design of multiplex sgRNA-tRNA array

Monocystronic tRNA sequences from *Clostridium pasteurianum* were complemented with 20 nt of their pre-tRNA sequence at their 3′end using the GtRNAdb—Genomic tRNA Database (RRID: SCR_006939) (Chan & Lowe, 2009; 2016). Individual tRNA structures were modelled with the online tool RNAfold (Gruber et al., 2008), and manually examined using a variety of parameters. The pre-tRNAs Thr-TGT-1-1 and fMet-CAT-1-1 were selected for (in decreasing order of importance) having a weak A-U rich stem-loop approximately 16 nt from the CCA-3′-end of the mature tRNA (Sekiya et al., 1979), for lacking a 3′ poly-U tail which could induce the termination of transcription, for not forming a strong DNA secondary structure with the binding sequence of the oligonucleotide primers used to synthesize the tRNA array during PCR amplification (Zuker, 2003), and for being associated with a codon in relatively high usage within *C. autoethanogenum* to avoid upsetting excessively the balance of its tRNA pool.

### 2.3 Conjugation in *Clostridium autoethanogenum*

Two days before mating, one 0.2 mL *C autoethanogenum* cryostock of 10% DMSO was thawed and inoculated into 2 mL of pre-reduced liquid YTF. Early the next day, each vector was transformed into 20 μL of *E. coli* sExpress and the *C. autoethanogenum* inoculum was subcultured into 4 mL of pre-reduced liquid YTF at starting OD 0.05. On the day of the mating, a single sExpress transformant colony of each construct was inoculated into 5 mL of room-temperature liquid LB + Cm + Kan until OD reached 0.2. 1 mL of culture was then centrifuged at 3,000 rcf for 3 min and washed in 0.5 mL PBS before being centrifuged again. The resulting pellet was finally resuspended into 0.2 mL of *C. autoethanogenum* culture and gently spread onto a YTF plate without antibiotics. 20 h later, 0.6 mL of PBS was vigorously spread over the mating plate to resuspend the cells and constitute the mating slurry. The mating slurry was normalized to 0.6 mL with PBS in a microcentrifuge tube, and 0.2 mL of it was finally transferred onto YTF + D-cyc + Tm + Th plates for selection of transconjugants and induction of Target-AID constructs. For the preliminary characterisation of Target-AID, the transconjugant colonies were subsequently transferred onto a YTF + D-cyc + Tm + Th+5-FOA in order to select for the *ΔpyrE* genotype.

### 2.4 Plasmid loss

After mutagenesis and colony PCR, plasmids were lost by re-streaking mutants on YTF plates without antibiotic, then patching 10 single colonies on YTF plates with and without Tm. Patches which failed to grow on Tm were considered to have lost their plasmid. They were then inoculated in liquid YTF with and without Tm to prepare cryopreservation in 10% pre-reduced DMSO once they reached OD600∼0.4 without antibiotics, if there was still no growth in liquid cultures complemented with thiamphenicol.

### 2.5 Estimation of mutagenesis efficiency

The efficiency of Target-AID mutagenesis was roughly estimated by calculating the proportion of colonies which survived exposure to 5 mM 5-FOA after induction on 5 mM theophylline. Subsequent mutagenesis efficiencies were only estimated from the Sanger sequencing results of five transconjugants colonies per construct.

### 2.6 Sanger sequencing

The targeted genes of fifteen *C. autoethanogenum* vFS36_TA_pyrE colonies and five *C. autoethanogenum* colonies conjugated with each of the other plasmids were screened by Sanger sequencing after a colony PCR with Q5^®^ DNA polymerase and purification with QIAquick PCR Purification Kit from Qiagen (Venlo, Netherlands). Each colony aliquot was first boiled in 40 μL sterile ddH2O for 10 min at 98°C and centrifuged at 2,500 RCF for 1 min before using 1 μL of supernatant as DNA template for the PCR. Sanger Sequencing was performed by Eurofins Genomics (Ebersberg, Germany) and sequencing results were aligned with their respective WT sequences using Benchling (RRID:SCR_013955). Sequencing primers are summarized in Supplementary Table S1 and raw reads are available in the Supplementary Material.

### 2.7 Determination of non-essentiality

Prior to plasmid design the three genes CLAU_532, CLAU_534 and CLAU_1794 were cross-checked in the published list of essential genes determined by transposon insertion sequencing (Woods et al., 2022) and confirmed to be non-essential under heterotrophic or autotrophic conditions.

### 2.8 Whole genome sequencing

Whole genome sequencing was performed at the Deepseq Next-Generation Sequencing Facility of the University of Nottingham.

#### 2.8.1 Library preparation, library QC and sequencing protocol

DNA concentration was measured using the Qubit Fluorometer and the Qubit dsDNA BR Assay Kit (ThermoFisher; Q32853). 250 ng input DNA was used in the sequencing library preparation. Indexed sequencing libraries were prepared using the Nextera DNA Flex Library Prep Kit (Illumina; 20018705) and IDT^®^ for Illumina Nextera™ DNA CD Indexes (Illumina; 20018708). 5 cycles of PCR were used. Libraries were quantified using the Qubit Fluorometer and the Qubit dsDNA HS Kit (ThermoFisher Scientific; Q32854). Library fragment-length distribution was assessed using the Agilent TapeStation 4,200 and the Agilent High Sensitivity D1000 ScreenTape Assay (Agilent; 5067-5584 and 5067-5585). Final library quantification was performed using the KAPA Library Quantification Kit for Illumina (Roche; KK4824) and the library was sequenced on an Illumina MiSeq using the MiSeq Reagent Kit v3 (600 cycle) (Illumina; MS-102-3,003) to generate 300-bp paired-end reads.

#### 2.8.2 Data analysis for resequencing

##### 2.8.2.1 Quality check and trimming of reads

Sequencing produced 1,919,451 raw reads that were trimmed of Illumina adapters and low quality (Q < 30) nucleotides using TrimGalore (v 0.6.6) (Andrews, 2010; Martin, 2011; Kreuger, 2012) nucleotide clip was performed at the 3′end of reads. Reads shorter than 20 bp were discarded. After quality filtering, 98.1% of reads remained.

##### 2.8.2.2 Alignment of reads to reference genome

Trimmed reads were aligned to the *Clostridium autoethanogenum* reference genome (NCBI accession: NZ_CP012395.1) using “bwa mem” (v0.7.17) (Li & Durbin, 2009; 2010; Li, 2013). Putative SNPs were filtered using “bcftools mpileup”, with minimum base and mapping quality of 20 applied and duplicate reads removed. SNPs were typed using ‘bcftools call’ with low quality genotypes filtered out (SNP quality <30 OR Mapping quality <30) (van der Auwera and O’Connor, 2020; Danecek et al., 2021). Mapping to the reference genome achieved coverage of 99.7% of reference bases at an average depth of 213x.

##### 2.8.2.3 Annotation of variants

Genome annotation associated with NZ_CP012395.1 was downloaded from the NCBI database and appropriate annotations added to variants.

#### 2.8.3 Identification of putative off-target mutations

All protospacers sequences targeted in cFS05 were submitted to Cas-Offinder (RRID: SCR_023390) to generate a list of putative off-target sites with up to 9 mismatches, 2 nt DNA gaps and 2 nt RNA gaps. This generated 14,376 putative off-target sites. Sites within 50 nt of the undesired SNP detected by whole genome sequencing were considered as potential off-targets of their associated protospacer. Additionally, the sequences of all protospacers targeted in cFS05 were aligned with the 41 nt WT sequence of each undesired SNP and the 20 nt immediately upstream and downstream in order to find potential matches between target protospacers and the sequence around undesired SNP using Geneious (RRID:SCR_010519).

## 3 Results

### 3.1 Target-AID proof-of-concept: *pyrE* knockout in *Clostridium autoethanogenum*

**FIGURE 2:**
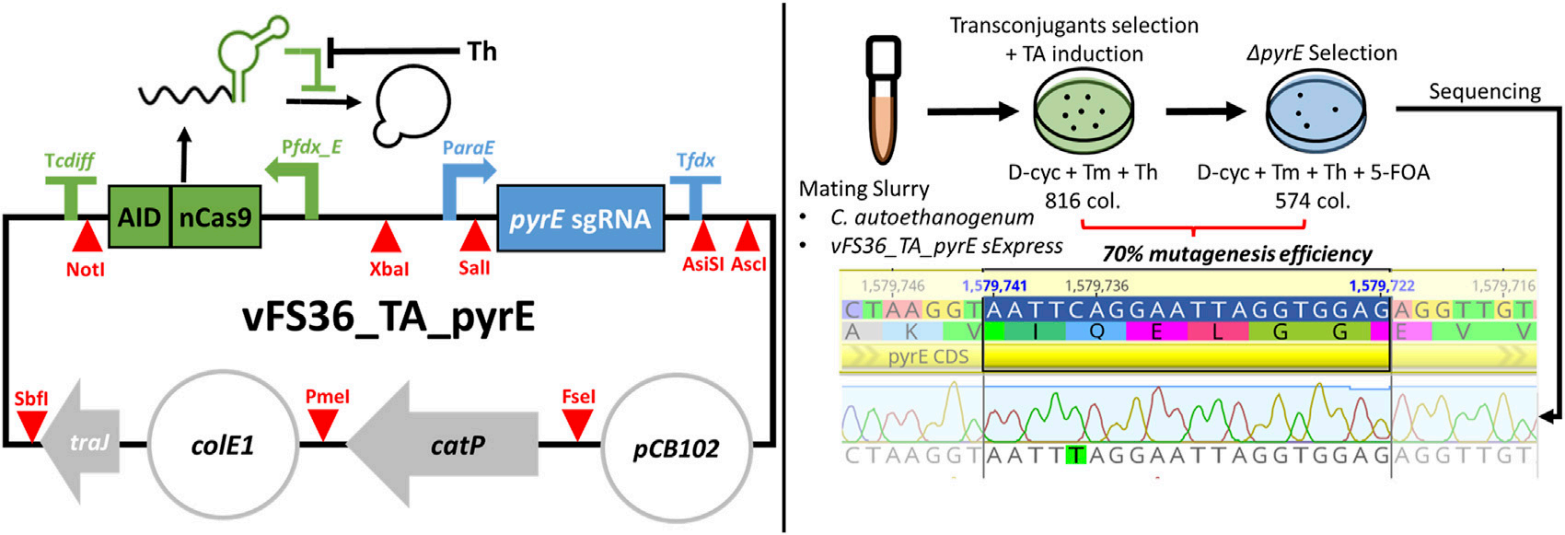
Target-AID proof of concept. (Left) Schematic of the plasmid vFS36_TA_pyrE, based on the pMTL83151 backbone. (Right) Knock-out of *pyrE* in *Clostridium autoethanogenum* using Target-AID. 816 colonies were obtained on the transconjugants selection plate with 5 mM Th; after transfer onto a plate complemented with 5 mM 5-FOA, only 574 colonies remained. The *pyrE* loci of fifteen 5-FOA-resistant colonies were then screened by Sanger sequencing, and all exhibited the desired C388T mutation. Blue highlighted region = 20 nt targeted protospacer; TA = Target-AID; D-cyc = D-cycloserine; Tm = Thiamphenicol; Th = Theophylline; 5-FOA = 5-Fluoroorotic acid; col. = colonies.Symbols inspired from SBOL visual (Baig et al., 2021).

The Target-AID design was validated with the plasmid vFS36_TA_pyrE (Figure 2), customised to introduce a premature STOP codon in the *pyrE* gene of *C. autoethanogenum*, which is necessary for pyrimidine synthesis and confers resistance to 5-FOA when knocked out (Ng et al., 2013; Minton et al., 2016). Only 70% of the 816 transconjugant colonies induced with theophylline survived when transferred on 5-FOA, but all the fifteen 5-FOA-resistant colonies screened exhibited the expected C388T mutation which results in a premature STOP codon (TAG) (Figure 2). This preliminary experiment confirmed that our basic Target-AID construct and mutagenesis protocol was functional in *C. autoethanogenum*.

### 3.2 Multiplexing designs

**FIGURE 3:**
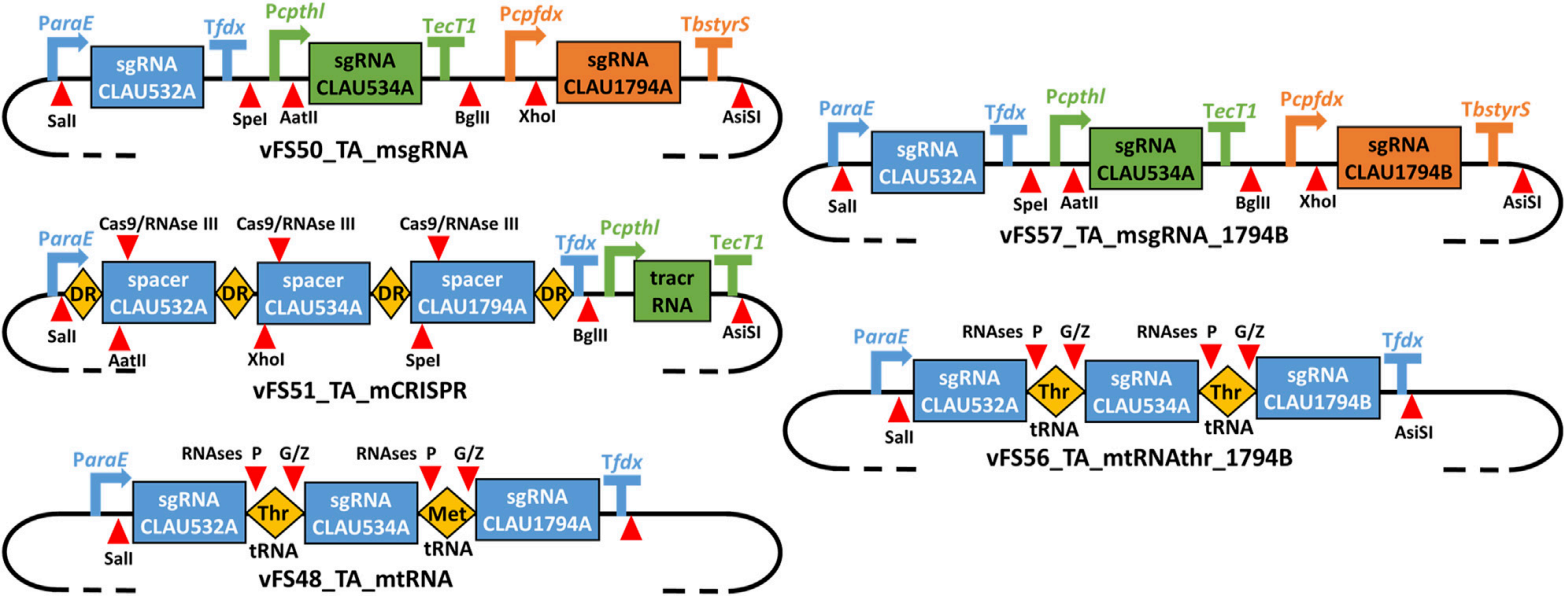
Schematic of the gRNA expression cassettes of different multiplex Target-AID plasmids. vFS50_TA_msgRNA has three separate sgRNA transcriptional units; vFS51_TA_mCRISPR has two transcriptional units expressing a CRISPR array and a tracrRNA, respectively; vFS48_TA_mtRNA, has a single transcriptional unit expressing an array of sgRNA-tRNA fusions All are derived from vFS36_TA_pyrE. vFS57_TA_msgRNA_1794B is derived from vFS50_TA_msgRNA and only differs in the protospacer sequence targeting CLAU_1794 (CLAU1794B instead of CLAU1794A). vFS56_TA_mtRNAthr_1794B is derived from vFS48_TA_mtRNA but uses twice the same tRNA (Thr-TGT-1-1 tRNA) and targets the CLAU1794B protospacer instead of CLAU1794A. DR = Direct repeat; tracrRNA = trans-activating CRISPR RNA. Symbols inspired from SBOL visual (Baig et al., 2021).

The msgRNA, mCRISPR and mtRNA multiplexing strategies were respectively tested by the plasmids vFS50_TA_msgRNA vFS51_TA_mCRISPR and vFS48_TA_mtRNA (Figure 3). Because only the last 20 nt at the 3′-end of the CRISPR spacer were shown to be necessary for gRNA targeting, but the native *S. pyogenes* CRISPR array is composed of ∼30 nt spacers (Jinek et al., 2012), each 20 nt protospacer sequence of vFS51_TA_mCRISPR was complemented with a 6 nt restriction site and 4 random nt at its 5′-end. Three non-selectable and non-essential genes (CLAU_532, CLAU_534 and CLAU_1794) coding for alcohol dehydrogenases were picked as arbitrary targets in *C. autoethanogenum*, and protospacers which could produce a STOP codon after a C-to-T mutation were identified for each gene (labelled CLAU532A, CLAU534A, and CLAU1794A, respectively).

In addition to these three multiplex constructs, three “monoplex” controls were also assembled to estimate the individual effectiveness of each individual sgRNA independently from its multiplexing system (Table 3). These are the vectors vFS52_TA_CA532A, vFS53_TA_CA534A, and vFS54_TA_CA1794A, all directly derived from vFS36_TA_pyrE with the *pyrE* protospacer sequence replaced by a protospacer sequence of their respective target gene.

**TABLE 3:** Sequencing of five *Clostridium autoethanogenum* colonies conjugated with different Target-AID constructs and induced on theophylline. vFS48 = vFS48_TA_mtRNA; vFS50 = vFS50_TA_msgRNA; vFS51 = vFS51_TA_mCRISPR; vFS56 = vFS56_TA_mTHRtRNA; vFS57 = vFS57_TA_msgRNA_CA1794B; vFS52 = vFS52_TA_CA532A; vFS53 = vFS53_TA_CA534A; vFS54 = vFS54_TA_CA1794A; and vFS58 = vFS58_TA_CA1794B. The targeted codon is bolded and mutated bases are capitalized. Fractions indicate mixed reads for this base. The sequence of CLAU532A is oriented in the antisense direction, meaning that TTA, TCA, and CTA alleles would all results in a STOP codon (TAA, TGA and TAG, respectively).

Protospacer	Allele	Sequence (20 nt)	Mutagenesis efficiency (over 5 colonies)								
			Multiplex constructs					Monoplex constructs			
			vFS48	vFS50	vFS51	vFS56	vFS57	vFS52	vFS53	vFS54	vFS58
CLAU532A	WT	ct**c​ca**g​tca​ggt​gtt​gtg​ca	0	1/5	4/5	0	0	0			
	A.1	ct**TTa**gt​cag​gtg​ttg​tgc​a	0	1/5	0	0	1/5	0			
	A.2	ct $\frac{T}{c}\frac{T}{c}$ **a**gtcaggtgttgtgca	0	3/5	0	0	0	5/5			
	A.3	ct $\frac{T}{c}$ **ca**gtcaggtgttgtgca	5/5	0	1/5	3/5	3/5	0			
	A.4	ct**Tca**g​tca​ggt​gtt​gtg​ca	0	0	0	2/5	0	0			
	A.5	ct**T** $\frac{T}{c}$ **a**gtcaggtgttgtgca	0	0	0	0	1/5	0			
CLAU534A	WT	agc​c**ca​a**tg​tct​agc​tgg​ga	5/5	0	5/5	3/5	0		2/5		
	A.1	agTT**Taa**t​gtc​tag​ctg​gga	0	0	0	0	0		0		
	A.2	agTc**ca​a**tg​tct​agc​tgg​ga	0	0	0	0	1/5		1/5		
	A.3	ag $\frac{T}{c}$ c**caa**tgtctagctggga	0	0	0	2/5	2/5		2/5		
	A.4	ag $\frac{T}{c}\frac{T}{c}\frac{T}{c}$ **aa**tgtctagctggga	0	5/5	0	0	0		0		
	A.5	ag $\frac{T}{c}\frac{T}{c}$ **caa**tgtctagctggga	0	0	0	0	1/5		0		
	A.6	agTT**caa**​tgt​cta​gct​ggg​a	0	0	0	0	1/5		0		
CLAU1794A	WT	aaa​**caa**​gca​att​gtt​ccg​tt	5/5	5/5	5/5					5/5	
	A.1	aaa**Taa**g​caa​ttg​ttc​cgt​t	0	0	0					0	
CLAU1794B	WT	atc​a**ca​a**tg​ttt​agc​agg​ta				3/5	3/5				1/5
	A.1	atTa**Taa**t​gtt​tag​cag​gta				0	0				0
	A.2	atTa**ca​a**tg​ttt​agc​agg​ta				0	1/5				2/5
	A.3	at $\frac{T}{c}$ a**caa**tgtttagcaggta				2/5	1/5				2/5

### 3.3 Most Target-AID mutants exhibit mixed genotypes

No mutants could be obtained from any of the gRNAs targeting the protospacer CLAU1794A (Table 3). However, CLAU532A and CLAU534A were successfully mutated by their respective monoplex control and vFS50_TA_msgRNA. Unfortunately, all but two of the twenty-three mutated loci showed some level of mixed trace, revealing the presence of WT cells in the colony. In most cases, the WT trace was dominant over the mutated one. Only one colony transformed with a multiplex construct (vFS50_TA_msgRNA) showed a pure colony with the desired mutation for a protospacer (CLAU532A.A1). Fortunately, it also showed a mixed peak for the CLAU534A protospacer (CLAU534A.A4). That colony was thus re-streaked on a YTF + D-cyc + Tm + Th plate to isolate pure colonies. A single pure colony with the desired CLAU532A.A1 and CLAU534A.A1 C-to-T mutations could be identified after screening five of these re-streaked colonies (cFS15). Albeit with poor efficiency, this is evidence that at least two loci can be mutated with premature STOP codon in a single mutagenesis step using Target-AID.

A subsequent multiplex mutagenesis attempt was made with a different CLAU_1794 protospacer (CLAU1794B) with the plasmid vFS57_TA_msgRNA_1794B (Figure 3) and its monoplex control vFS58_TA_CA1794B. This time, the last protospacer could be targeted, albeit only two out of five colonies showed a mutated CLAU1794B protospacer (four for the monoplex control), both showed a mixed trace, and none had the desired C-to-T mutation which would have produced a STOP codon. This confirmed that the last sgRNA cassette was functional and that the previous failure to mutate CLAU1794A was not due to any multiplex system, but to a defective protospacer sequence. Unfortunately, the CLAU534A.A1 or CLAU534A.A4 allele could not be found among the five screened vFS57_TA_msgRNA_1794B colonies.

### 3.4 Arrays of tRNA-sgRNA fusions can be used to express multiple sgRNAs

The initial mtRNA and the mCRISPR multiplexing strategies both failed to mutate the CLAU534A protospacer, even though this could be achieved by the msgRNA strategy and the monoplex control targeting CLAU534A (vFS53_TA_CA534A). The mCRISPR strategy only managed to mutate one of the five colonies screened for CLAU532A mutations (Table 3). Interestingly the mtRNA strategy managed to mutate all five of them. This indicated that the Thr-TGT-1-1 tRNA had not interfered with the function of the CLAU532A sgRNA. The mCRISPR strategy was abandoned at this stage. However, we hypothesized that replacing the fMet-CAT-1-1 tRNA by another copy of the Thr-TGT-1-1 tRNA might rescue the mtRNA strategy.

While multiplex mutagenesis with the vFS56_TA_mtRNAthr_1794B (Figure 3), which only uses the Thr-TGT-1-1 tRNA, did not yield the expected C-to-T mutation in either CLAU534A or CLAU1794B, it did successfully target all three protospacers and even yielded two pure CLAU532A.A4 alleles with the desired C-to-T mutation (Table 3).

### 3.5 UGI-LVA and truncated sgRNAs do not improve mutagenesis efficiency

Next, we investigated whether fusing a UGI (with GLVA degradation tag) to Target-AID would improve mutagenesis efficiency in all protospacers, or if truncating the protospacer region of the sgRNAs targeting CLAU534A and CLAU1794B would shift their editing window to edit the cytosine in position −16 from the PAM more favourably. Unfortunately, while the initial vFS57_TA-msgRNA_CA1794B construct once again managed to edit all three loci with almost 100% efficiency (although without pure mutant and only one correctly edited base out of ten in position −16 for CLAU534A and CLAU1794B), the addition of UGI-LVA downstream at the C-terminus of Target-AID in the plasmid vFS75_mTA-UGILVA seemed only to harm editing efficiency (only 2 pure mutations for CLAU534A and no other mutation across the 12 remaining reads), and truncating the sgRNAs targeting CLAU534A and CLAU1794B from 20 nt to 18 nt in the plasmid vFS94_mTA-trsgRNA seemed to completely abolish mutagenesis of their respective protospacers (Table 4).

**TABLE 4:** Sequencing of five *Clostridium autoethanogenum* colonies conjugated with different multiplex Target-AID constructs and induced on theophylline. vFS75_mTA-UGI-GLVA is a msgRNA Target-AID construct fused with a UGI and GLVA domains at its C-terminus. vFS94_mTA-trsgRNA is a msgRNA Target-AID construct with 18 nt spacers instead of 20 nt. vFS57_TA-msgRNA_CA1794B is a standard msgRNA Target-AID construct (without UGI-GLVA tag and with 20 nt spacers) that targets the same protospacers as vFS75_mTA-UGI-GLVA and vFS94_mTA-trsgRNA. The targeted codon is bolded and mutated bases are capitalized. Fractions indicate mixed reads for this base. The sequence of CLAU532A is oriented in the antisense direction, meaning that TTA, TCA, and CTA alleles would all results in a STOP codon (TAA, TGA and TAG, respectively). (*) Poor quality reads were removed from analysis.

Protospacer	Allele	Sequence (20 nt)	Mutagenesis efficiency (over 5 colonies)		
			Redesigned		Control
			vFS75_mTA-UGI-GLVA	vFS94_mTA-trsgRNA	vFS57_TA-msgRNA_CA1794B
CLAU532A	WT	ct**c​ca**g​tca​ggt​gtt​gtg​ca	4/4*	0	0/5
	A.2	ct $\frac{T}{c}\frac{T}{c}$ **a**gtcaggtgttgtgca	0	0	5/5
	A.3	ct $\frac{T}{c}$ **ca**gtcaggtgttgtgca	0	4/5	0
	A.6	Tt $\frac{T}{c}\frac{T}{c}$ **a**gtcaggtgttgtgca	0	1/5	0
CLAU534A	WT	agc​c**ca​a**tg​tct​agc​tgg​ga	3/5	5/5	0
	A.2	agTc**ca​a**tg​tct​agc​tgg​ga	2/5	0	0
	A.5	ag $\frac{T}{c}\frac{T}{c}$ **caa**tgtctagctggga	0	0	1/5
	A.7	agT $\frac{T}{c}$ **caa**tgtctagctggga	0	0	3/5
	A.8	agT $\frac{T}{c}\frac{T}{c}$ **aa**tgtctagctggga	0	0	1/5
CLAU1794B	WT	atc​a**ca​a**tg​ttt​agc​agg​ta	5/5	5/5	1/5
	A.3	at $\frac{T}{c}$ a**caa**tgtttagcaggta	0	0	4/5

### 3.6 Target-AID-NG has a vastly superior targeting space

**FIGURE 4:**
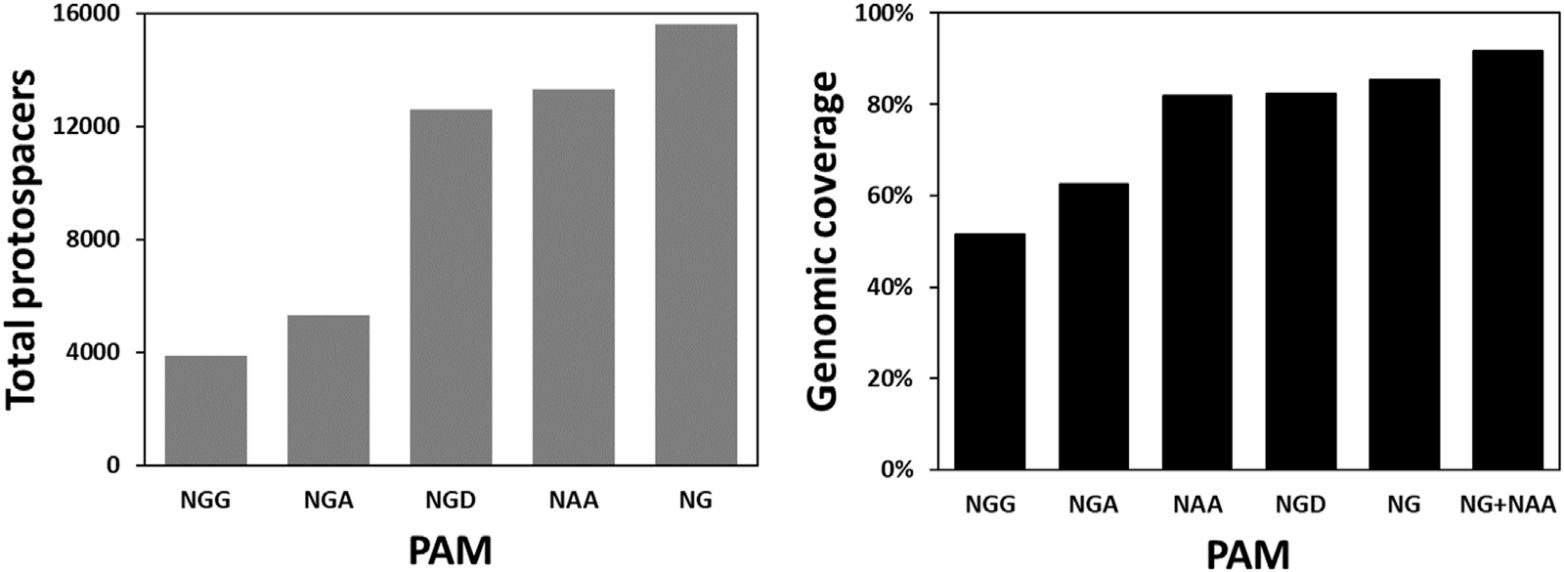
Targeting space and genomic coverage of Target-AID in *C. autoethanogenum* as a function of different PAM sequences. The targeting space is defined here as the total number of protospacers in the first 75% of their respective CDS which can lead to a STOP codon when the cytosines within the nucleotides −19 to −16 from the PAM are mutated into thymines. We define genomic coverage as the proportion of CDS’s in the genome which have at least one of these protospacers.

Besides CLAU534A, CLAU1794A and CLAU1794B, there were no other Target-AID-compatible protospacers that could potentially result in a premature STOP codon in CLAU_534 or CLAU_1794. This revealed an inherent weakness of Target-AID mutagenesis: very few protospacers are available for each gene, and many genes cannot be targeted at all. To quantify the problem, the total number of Target-AID protospacers which could produce STOP codons in the first 75% of any gene in *C. autoethanogenum* were modelled and compared across several published SpCas9 variants (Supplementary Table S6) which exploit non-canonical PAMs (Figure 4). The proportion of *C. autoethanogenum* genes which could theoretically be disrupted by Target-AID using these protospacers for each PAM (Figure 4) was also measured. We named this parameter the genomic coverage of Target-AID.

The bioinformatic analysis undertaken revealed that the conventional Target-AID, with its NGG PAM, could potentially introduce STOP codons in only 3,895 protospacers for a genomic coverage of merely 51.64% in *C. autoethanogenum*. NG PAMs (Nishimasu et al., 2018), on the other hand, would give access to 15,602 protospacers which would theoretically allow 85.32% of *C. autoethanogenum*’s CDS to be inactivated. Also promising is the NAA PAM of Cas9-iSpymac (Chatterjee et al., 2020), which can target 13,293 protospacers for a genomic coverage of 81.81%. Together, Target-AID-NG and Target-AID-iSpymac would be able to knock out 91.78% *C. autoethanogenum*’s CDS. However, since both CLAU_532 and CLAU_534 happen to be among the 18.19% of CDS that Cas9-iSpymac could not knock-out as part of a Target-AID base editor, Target-AID-NG was chosen for a last multiplex mutagenesis attempt of CLAU_532, CLAU_534 and CLAU_1794 with the msgRNA strategy.

### 3.7 UGI without LVA tag does not improve mutagenesis efficiency of Target-AID-NG in *Clostridium autoethanogenum*

The vectors vFS72_mTA-NG and vFS103_mTA-NG-UGI_NoLVA were assembled to introduce nonsense mutations in CLAU_532, CLAU_534 and CLAU_1794 using Target-AID-NG and Target-AID-NG-UGI without GLVA tag (Table 5). Our model showed that Target-AID-NG could target five protospacers in CLAU_532, three in CLAU534, and seven in CLAU1794. This allowed the selection of protospacers where all the cytosines in the editing window would result in a nonsense mutation if mutated to thymines, with a preference for protospacers with cytosines in position −18 rather than −16 from the PAM. This time, with a UGI fusion but without GLVA tag, mutants were obtained at roughly the same rate as the Target-AID-NG construct without UGI (80% and 100% for CLAU532A, 0% and 20% for CLAU534B, and 80% and 60%, respectively). Only two traces out of 28 reflected pure mutants for one locus; all the other mutated loci were mixed with WT genotype (often predominantly WT). After one round of re-streak, no CLAU532A mutant could be isolated from the two colonies exhibiting the pure CLAU1794C.A.1 genotype.

**TABLE 5:** Sequencing of five *Clostridium autoethanogenum* colonies conjugated with different Target-AID-NG constructs and induced on theophylline. vFS72 = vFS72_mTA-NG; vFS103 = vFS103_mTA-NG-UGI_NoLVA. The targeted codon is bolded and mutated bases are capitalized. Fractions indicate mixed reads for this base. The sequence of CLAU532A and CLAU1794C is oriented in the antisense direction, meaning that TTA, TCA, and CTA alleles would all results in a STOP codon (TAA, TGA and TAG, respectively). (*) Poor quality reads were removed from analysis.

Protospacer	Allele	Sequence (20 nt)	Mutagenesis efficiency (over 5 colonies)	
			vFS72_mTA-NG	vFS103_mTA-NG-UGI_NoLVA
CLAU532A	WT	ct**c​ca**g​tca​ggt​gtt​gtg​ca	1/5	0
	A.2	ct $\frac{T}{c}\frac{T}{c}$ **a**gtcaggtgttgtgca	3/5	0
	A.3	ct $\frac{T}{c}$ **ca**gtcaggtgttgtgca	1/5	3/3*
	A.8	Tt $\frac{T}{c}\frac{T}{c}$ **a**gtcaggtgttgtgca	0	0
CLAU534B	WT	agg​**cag**​aag​gca​caa​ttt​gt	5/5	4/5
	A.1	agg $\frac{T}{c}$ **ag**aaggcacaatttgt	0/5	1/5
CLAU1794C	WT	at**c​ca**a​tct​ggc​cca​aat​tc	1/5	2/5
	A.1	at**TTa**at​ctg​gcc​caa​att​c	2/5	0
	A.2	at**T** $\frac{T}{c}$ **a**atctggcccaaattc	2/5	0
	A.5	at $\frac{T}{c}$ **ca**atctggcccaaattc	0	3/5

### 3.8 Target-AID-NG is also suitable for duplex mutagenesis in *Clostridium autoethanogenum*

In a last attempt to mutate all three targeted genes in the same strain, we assembled the vector vFS74_TA_NG_msgRNA_1794DFG to target three of the four remaining CLAU_1794 protospacers targetable by Target-AID-NG to produce a nonsense mutation. This vector was not using a UGI fusion and was using the msgRNA strategy. It was conjugated into the plasmid-free double-mutant *C. autoethanogenum* strain (cFS04) that had been previously engineered with vFS50_TA_msgRNA. As described in Table 6, the protospacer CLAU1794D failed to deliver any mutations in the five colonies screened, but CLAU1794F and CLAU1794G showed 100% of mutagenesis efficiency. This time, only three out of ten traces showed mixed peaks, but all five pure CLAU1794F mutations affected a cytosine which did not result in the introduction of a STOP codon. Out of the three protospacers, only CLAU1794G resulted in nonsense mutations in CLAU_1794.

**TABLE 6:** Sequencing of five *Clostridium autoethanogenum* colonies conjugated with vFS74_TA_NG_msgRNA_1794DFG and induced on theophylline. The targeted codon is bolded and mutated bases are capitalized. Fractions indicate mixed reads for this base. The sequence of CLAU1794G is oriented in the antisense direction, meaning that TTA, TCA, and CTA alleles would all result in a STOP codon (TAA, TGA and TAG, respectively).

Protospacer	Allele	Sequence	Mutagenesis efficiency (over 5 colonies)
			vFS74_mTA-NG_CA1794DFG
CLAU1794D	WT	Aga​**caa**​aaa​gct​aaa​ttt​gt	5/5
	A.1	Aga**Taa**a​aag​cta​aat​ttg​t	0/5
CLAU1794F	WT	tca​**caa**​tgt​tta​gca​ggt​at	0
	A.1	tca**Taa**t​gtt​tag​cag​gta​t	0
	A.2	tTa**ca​a**tg​ttt​agc​agg​tat	5/5
CLAU1794G	WT	g**cc​a**ta​cag​ctc​ctg​ttt​ta	0
	A.1	g**TTa**ta​cag​ctc​ctg​ttt​ta	1/5
	A.2	g**Tca**t​aca​gct​cct​gtt​tta	1/5
	A.3	g $\frac{T}{c}$ **ca**tacagctcctgtttta	3/5

**TABLE 7:** Summary of off-target SNP and short duplication events identified in *Clostridium autoethanogenum* after two rounds of multiplex Target-AID (then Target-AID-NG) mutagenesis, and their putative association with targeted protospacers. (*) Found by standard alignment with the protospacers targeted in this strain (CLAU532A, CLAU534A, CLAU1794A, CLAU1794D, CLAU1794F, CLAU1794G) using a standard alignment tool (Geneious (RRID:SCR_010519)), instead of Cas-Offinder (RRID: SCR_023390) (Zhao et al., 2017).

Locus	Position	WT allele	Alternate allele	Putative protospacer	Mismatch (nt)	Gap (nt)	CAS-OFFINDER position (nt)
CLAU_0577	660931	C	T	CLAU1794G	9	1	660941
CLAU_0894	995262	C	T	N/A			
CLAU_1083	1205885	C	A	CLAU532A	9	0	1205894
CLAU_1087	1210587	C	A	N/A			
CLAU_1384	1525791	G	A	CLAU534A	6	2	1525752
CLAU_1943	2136051	T	C	N/A			
CLAU_2237	2449821	A	C	CLAU1794G	9	0	2449824*
CLAU_2237	2450037	G	T	CLAU1794D	6	2	2450048
CLAU_2344	2570391	TCCTGAAAGGACATCA	TCC​TGA​AAG​GAC​ATC​ACC​TGA​AAG​GAC​ATC​A	N/A			
N/A	2994056	G	A	N/A			
CLAU_3637	4014166	C	T	N/A			
CLAU_3830	4203465	T	G	CLAU534A	8	2	4203417
CLAU_3935	4318262	G	A	CLAU534A	7	2	4318245

### 3.9 Fifteen off-target mutations were identified after two rounds of Target-AID and target-AID-NG multiplex mutagenesis

The resulting triple-mutant strain was finally validated by whole genome sequencing (NCBI accession PRJNA956560). It revealed twelve off-target single-nucleotide polymorphisms (SNP) (Supplementary Table S7): six exhibiting the canonical Target-AID C-to-T or G-to-A mutation, six within 50 nt of a putative off-target protospacer identified with Cas-Offinder (RRID: SCR_023390) (Zhao et al., 2017), and one which could be manually aligned with the protospacer CLAU1794G using Benchling (RRID:SCR_013955) sequence alignment tool (Supplementary Table S8). One 15 nt duplication event was also found, in addition to two regions of the genomes where the sequencing coverage abruptly dropped to 0 and thus seem to have been lost by the cell (Table 8). One of these deleted regions was only 23 nt long (but inside one of the nine 16S RNA loci), while the other region was 12 kb-long and comprised a putative off-target protospacer relatively similar to its on-target, with only four mismatches and a 2 nt gap. Altogether, fifteen off-target mutations were thus identified in our strain that underwent two consecutive rounds of multiplex Target-AID (then Target-AID-NG) mutagenesis. Importantly, the gene coding for UDG was among the thirteen genes lost within the 12 kb deleted region (Table 8; Supplementary Table S9 for detailed list of deleted genes).

**TABLE 8:** Summary of gene deletion events identified in *Clostridium autoethanogenum* after two rounds of multiplex Target-AID (then Target-AID-NG) mutagenesis, and their putative association with targeted protospacers. Putative off-target protospacers identified with Cas-Offinder (RRID: SCR_023390) (Zhao et al., 2017).

Coverage = 0					
Start position	Size (nt)	Putative protospacers	Mismatches (nt)	Gap (nt)	Cas-offinder position (nt)
2360221	12053	1794G	4	2	2372289
3873128	23	N/A			

## 4 Discussion

Our results show that, although Target-AID and Target-AID-NG can be used for multiplexed targeted point mutagenesis in *C. autoethanogenum*, they have serious drawbacks. While mutagenesis efficiency is high enough to reliably isolate mutants without selection markers, the resulting colonies are often mixed, which requires additional re-streaking steps to isolate pure mutants. Mixed colonies have been reported previously with several base editors in different organisms (Banno et al., 2018; Li et al., 2019; Liu et al., 2019). We hypothesize that they result from single cells that initially survived Target-AID mutagenesis by undergoing BER or by replicating instead of mutating their non-edited strand (Figure 1.VI, 1.VIII). From there, one of the cell lineages from the colony can mutate and produce the expected mutation, while the second lineage of cells continues to avoid mutagenesis by a variety of mechanisms, for example, by mutating key components of the Target-AID plasmid. The same process could produce mixed colonies when more than one cytosines are present in the editing window: each cell lineage in the same colony would acquire immunity from Target-AID by mutating a different cytosine. Protospacers with multiple cytosines in the editing window should thus be avoided to minimize the risk of obtaining mixed colonies.

Targeting cytosines in position −16 or −17 from the PAM, while possible, was rarely successful—especially when other cytosines were present in positions −18 or −19. Accordingly, only protospacers with a single cytosine in position −18 or −19 from the PAM should be used. This is consistent with the literature (Banno et al., 2018; Li et al., 2019; Nishida et al., 2016), but it severely restricts the already limited range of available protospacers in *C. autoethanogenum*. As illustrated in this work, the use of base editors with alternative PAM recognition domains such as Target-AID-NG or Target-AID-iSpymac can, however, greatly facilitate the identification of optimal protospacer targets.

This feature of Target-AID-NG was exploited to test three protospacers at once for the same target gene, in the hope that at least one would work. Indeed, out of the ten targeted protospacers (*pyrE* protospacer, CLAU532A, CLAU534A-B, and CLAU1794A-B-C-D-F-G), two did not work at all (CLAU1794A, and CLAU1794D) and only four reliably introduced nonsense mutations (*pyrE* protospacer, CLAU532A, CLAU1794C, and CLAU1794G). Targeting the same gene with several protospacers at once might thus still have been a good use of multiplexing and Target-AID, if it had not also been associated with such a high rate of off-target mutagenesis (including a major 12 kb deletion). Consequently, even though we showed multiplex Target-AID mutagenesis was achievable in *C. autoethanogenum*, we cannot recommend its use as a standard practice, even just to screen several protospacers for the same target gene. Using parallel monoplex constructs is indeed less likely to result in off-target mutagenesis for the same number of targeted genes.

It is, however, difficult to assert with confidence which off-target mutations are a direct consequence of Target-AID mutagenesis. Some probably occurred randomly and were only selected through the many rounds of re-streaking; others might have been the indirect consequence of the loss of UDG, a key enzyme of the BER DNA repair pathway. The loss of UDG itself, nonetheless, was likely selected for by Target-AID, as UDG is a putative inhibitor of CBEs mutagenesis and thus a liability for the cells which express Target-AID. We hypothesize that UDG was lost in a random recombination event (potentially triggered by an off-target nick from Target-AID). *Δudg* cells should lose the BER pathway, which might have made them more susceptible to Target-AID mutagenesis. Accordingly, the *Δudg* mutants must have been over-represented in the colonies that survived mutagenesis. After the MMR pathway mutated the non-edited strand, in the absence of UDG, the repair of the edited strand from a “U” to a “T” might have been achieved solely through DNA replication.

The loss of UDG indirectly validates the strategy of using an UGI fusion to improve the effectiveness of CBEs, but it also exposes the gene coding for UDG as a mutational hotspot to look out for during Target-AID (or CBE) mutagenesis. This result highlights the importance of whole genome sequencing and rigorous complementation studies in any genome editing experiment, including base editing. Interestingly, in our hands, truncated sgRNA spacers, Target-AID-UGI and Target-AID-UGI-GLVA protein fusions did not result in higher mutagenesis efficiencies in *C. autoethanogenum* (there was even a marked decrease of mutagenesis efficiency when the GLVA-tag was present or truncated sgRNA spacers were used).

Here, multiplexing gRNA transcription using the native CRISPR array of *S. pyogenes* was not successful. In hindsight, it might be due to the addition of a restriction site and four extra nt upstream of each 20 nt spacer to facilitate cloning. Future iterations of this multiplexing strategy would be advised to comprise of 30 nt spacers fully homologous to their target. The tracrRNA could also be flanked with self-cleaving ribozymes to exclude any interference from its promoter and terminator in its RNA structure.

While an investigation of whether the tRNA-sgRNA array had successfully been processed into individual tRNAs and sgRNAs molecules was not undertaken, the array succeeded in targeting multiple protospacers almost as effectively as three separate transcriptional units. Given that the monoplex controls also failed to produce the desired nonsense mutation, the absence of correct CLAU534A and CLAU1794B mutants in either multiplexing strategy can be attributed to the protospacers themselves, and not the multiplexing method. An array of tRNA-sgRNA fusions has several advantages over an array of separate sgRNA transcriptional units. Notably, the reduced size of the sgRNA expression cassette (821 nt for mtRNA *versus* 1,146 nt for msgRNA) and its simplicity and scalability: only one promoter and terminator are needed to express an arbitrary number of sgRNA-tRNA fusions. Beyond Cas9 mutagenesis, tRNA fusions which exploit prokaryotic tRNA maturation mechanisms to process polycistronic RNA into individual molecules could be used to express any RNA-based synthetic biology tool (Altman, 1975; Apirion & Miczak, 1993; Gegenheimer & Apirion, 1981; Green et al., 2014; Lee et al., 2018; Li et al., 2005; Li & Deutscher, 2002; Mackie, 2013; Minagawa et al., 2004; Mörl & Marchfelder, 2001; Ow & Kushner, 2002; Sekiya et al., 1979). This strategy could be improved by identifying more prokaryotic tRNAs which are compatible with sgRNAs arrays, to avoid repeating the same tRNA sequence in the same sgRNA array.

Finally, Target-AID is an exceedingly easy system to build, that does not require any PCR amplification step: just swapping the 20 nt protospacer in a single two-parts Gibson or Golden-Gate assembly between a cut vector and a synthesised DNA oligo is sufficient to create a vector capable of editing a different target. The absence of homology cassette makes it uniquely straightforward to multiplex, especially with polycistronic RNA systems such as the mtRNA or mCRISPR strategies illustrated in this study. However, the time gained during design and assembly can be quickly lost again if mixed colonies need to be re-streaked (especially for slow-growing organisms like *C. autoethanogenum*). Initial screening of mutants is also more difficult than with standard homology-directed knockouts (Seys et al., 2020), as a colony PCR cannot readily identify the desired SNPs through simple gel electrophoresis.

## 5 Conclusion

This study is valuable because it illustrates the strengths and weaknesses of the base editor Target-AID-NG in the *Clostridium* genus and exemplifies the use of prokaryotic tRNAs to process a synthetic polycistronic RNA.

Like other base editors, Target-AID-NG offers several undeniable advantages besides bypassing the need for a functional HR or NHEJ DNA repair pathway. It is straightforward to assemble and use in a standard mutagenesis workflow and can easily be multiplexed. However, it suffers from a restricted pool of optimal and/or functional protospacers, as well as a propensity to generate mixed colonies of WT and mutated cells. Critically, like any other genome editing tool to date, Target-AID-NG is still not precise or innocuous enough to ignore the possibility of off-target mutations and the necessity of complementation studies. As illustrated in this study, particular attention should be paid to the *udg* homolog of the targeted organism, as its loss might be selected by Target-AID-NG. With whole-genome sequencing technology becoming more affordable every year, it should become a standard step to any mutant characterisation.

Fortunately, base editing is a quickly evolving field (Anzalone et al., 2020; Kantor et al., 2020). While Target-AID or Target-AID-NG in their current form might find some niche applications in contexts where conventional HR- or NHEJ-mediated mutagenesis methodologies are impossible, the obstacles encountered in the present study make it difficult to recommend as a tool for mainstream knock-out experiments in *C. autoethanogenum* when other methods are available. Other base editors such as BE4 (Gaudelli et al., 2017; Gehrke et al., 2018; Li et al., 2019) or Target-AID-iSPymac (Chatterjee et al., 2020) might have marginally improved performances while conserving its simplicity of design and potential for multiplexing. Alternatively, the recently developed prime editor (Anzalone et al., 2019), that uses a reverse-transcriptase fused to nCas9 to introduce custom small mutations independently from the target sequence, might hold the greatest promise for a multiplex and scarless point mutagenesis tool in *Clostridium*.

## Data Availability

The datasets presented in this study can be found in online repositories. This data can be found here: [https://www.ncbi.nlm.nih.gov/bioproject/PRJNA956560]

## Abbreviations

ABE adenine base editors; AID activation-induced cytidine deaminase; BER Base excision repair; Cas CRISPR-associated protein; CBE cytosine base editors; CDS coding sequences; CRISPR clustered regularly interspersed repeats; CDA cytosine deaminase; Cm chloramphenicol; D-cyc D-cycloserine; DR direct repeat; DSB double-strand breaks; 5-FOA 5-Fluoroorotic acid; HR homologous recombination; Kan kanamycin; LB Luria-Bertani broth; nCas9 Cas9 nickase; NHEJ non-homologous end-joining; kb kilobase; nt nucleotide; MMR mismatch repair; mCRISPR multiplexing with a synthetic *S. pyogenes* CRISPR array; msgRNA multiplexing with multiple sgRNA expression cassettes; mtRNA multiplexing with sgRNA-tRNA fusions mtRNA; NEB New England Biolabs Ltd.; PAM protospacer adjacent motifs; PCR polymerase chain reactions; SNP single-nucleotide polymorphism; Th theophylline; Tm thiamphenicol; tracrRNA trans-activating CRISPR RNA; UDG uracil DNA glycosylase; UGI uracil glycosylase inhibitor; YTF yeast tryptone fructose.

## References

[B1] Adiego-Pérez B. Randazzo P. Daran J. M. Verwaal R. Roubos J. A. Daran-Lapujade P. (2019). Multiplex genome editing of microorganisms using CRISPR-Cas. FEMS Microbiol. Lett. 366 (8), fnz086–19. 10.1093/femsle/fnz086 31087001 PMC6522427

[B2] Altman S. (1975). Biosynthesis of transfer RNA in Escherichia coli. Cell 4 (1), 21–29. 10.1016/0092-8674(75)90129-4 1090377

[B3] Andrews S. (2010). FastQC: A quality control tool for high throughput sequence data. Available at: http://www.Bioinformatics.Babraham.Ac.Uk/Projects/Fastqc.

[B4] Anzalone A. v. Koblan L. W. Liu D. R. (2020). Genome editing with CRISPR–Cas nucleases, base editors, transposases and prime editors. Nat. Biotechnol. 38(7), 824–844. 10.1038/s41587-020-0561-9 32572269

[B5] Anzalone A. v. Randolph P. B. Davis J. R. Sousa A. A. Koblan L. W. Levy J. M. (2019). Search-and-replace genome editing without double-strand breaks or donor DNA. Nature 576 (7785), 149–157. 10.1038/s41586-019-1711-4 31634902 PMC6907074

[B6] Apirion D. Miczak A. (1993). RNA processing in prokaryotic cells. BioEssays 15(2), 113–120. 10.1002/bies.950150207 7682412

[B7] Baig H. Fontanarossa P. McLaughlin J. Scott-Brown J. Vaidyanathan P. Gorochowski T. (2021). Synthetic biology open language visual (SBOL visual) version 3.0. J. Integr. Bioinforma. 18 (3), 20210013. 10.1515/jib-2021-0013 PMC8560346 34668358

[B8] Banno S. Nishida K. Arazoe T. Mitsunobu H. Kondo A. (2018). Deaminase-mediated multiplex genome editing in Escherichia coli. Nat. Microbiol. 3 (4), 423–429. 10.1038/s41564-017-0102-6 29403014

[B9] Bourgade B. Minton N. P. Islam M. A. (2021). Genetic and metabolic engineering challenges of C1-gas fermenting acetogenic chassis organisms. Oxf. Univ. Press 45 (2), fuab008. 10.1093/femsre/fuab008 PMC8351756 33595667

[B10] Cañadas I. C. Groothuis D. Zygouropoulou M. Rodrigues R. Minton N. P. (2019). RiboCas: A Universal CRISPR-based editing tool for Clostridium. ACS Synth. Biol. 8 (6), 1379–1390. 10.1021/acssynbio.9b00075 31181894

[B11] Chan P. P. Lowe T. M. (2016). GtRNAdb 2.0: An expanded database of transfer RNA genes identified in complete and draft genomes. Nucleic Acids Res. 44 (D1), D184–D189. 10.1093/nar/gkv1309 26673694 PMC4702915

[B12] Chan P. P. Lowe T. M. (2009). GtRNAdb: A database of transfer RNA genes detected in genomic sequence. Nucleic Acids Res. 37, D93–D97. 10.1093/nar/gkn787 18984615 PMC2686519

[B13] Chatterjee P. Lee J. Nip L. Koseki S. R. T. Tysinger E. Sontheimer E. J. (2020). A Cas9 with PAM recognition for adenine dinucleotides. Nat. Commun. 11 (1), 2474. 10.1038/s41467-020-16117-8 32424114 PMC7235249

[B14] Danecek P. Bonfield J. K. Liddle J. Marshall J. Ohan V. Pollard M. O. (2021). Twelve years of SAMtools and BCFtools. GigaScience 10 (2), giab008. 10.1093/gigascience/giab008 33590861 PMC7931819

[B15] Davis M. W. Jorgensen E. M. (2022). Frontiers in Bioinformatics, 2. 10.3389/fbinf.2022.818619 ApE, A plasmid editor: A freely available DNA Manipulation and Visualization Program PMC9580900 36304290

[B16] Dong F. Xie K. Chen Y. Yang Y. Mao Y. (2017). Polycistronic tRNA and CRISPR guide-RNA enables highly efficient multiplexed genome engineering in human cells. Biochem. Biophysical Res. Commun. 482 (4), 889–895. 10.1016/j.bbrc.2016.11.129 PMC5284736 27890617

[B17] Eid A. Alshareef S. Mahfouz M. M. (2018). CRISPR base editors: Genome editing without double-stranded breaks. Biochem. J. 475 (11), 1955–1964. 10.1042/BCJ20170793 29891532 PMC5995079

[B18] Fackler N. Heijstra B. D. Rasor B. J. Brown H. Martin J. Ni Z. (2021). Stepping on the gas to a Circular Economy: Accelerating development of Carbon-negative chemical production from gas fermentation. Annu. Rev. Chem. Biomol. Eng. 12 (1), 439–470. 10.1146/annurev-chembioeng-120120-021122 33872517

[B19] Fukui K. (2010). DNA mismatch repair in eukaryotes and bacteria. J. Nucleic Acids 2010, 1–16. 10.4061/2010/260512 PMC2915661 20725617

[B20] Gaudelli N. M. Komor A. C. Rees H. A. Packer M. S. Badran A. H. Bryson D. I. (2017). Programmable base editing of A•T to G•C in genomic DNA without DNA cleavage. Nature 551 (7681), 464–471. 10.1038/nature24644 29160308 PMC5726555

[B21] Gegenheimer P. Apirion D. (1981). Processing of procaryotic ribonucleic acid. Microbiol. Rev. 45 (4), 502–541. 10.1128/mr.45.4.502-541.1981 6173734 PMC281526

[B22] Gehrke J. M. Cervantes O. Clement M. K. Wu Y. Zeng J. Bauer D. E. (2018). An apobec3a-cas9 base editor with minimized bystander and off-target activities. Nat. Biotechnol. 36 (10), 977–982. 10.1038/nbt.4199 30059493 PMC6181770

[B23] Green A. A. Silver P. A. Collins J. J. Yin P. (2014). Toehold switches: De-novo-designed regulators of gene expression. Cell 159 (4), 925–939. 10.1016/j.cell.2014.10.002 25417166 PMC4265554

[B24] Gruber A. R. Lorenz R. Bernhart S. H. Neubock R. Hofacker I. L. (2008). The Vienna RNA Websuite. Nucleic Acids Res. 36, W70–W74. 10.1093/nar/gkn188 18424795 PMC2447809

[B25] Huang H. Chai C. Li N. Rowe P. Minton N. P. Yang S. (2016). CRISPR/Cas9-Based efficient genome editing in Clostridium ljungdahlii, an autotrophic gas-fermenting bacterium. ACS Synth. Biol. 5 (12), 1355–1361. 10.1021/acssynbio.6b00044 27276212

[B26] Humphreys C. M. Mclean S. Schatschneider S. Millat T. Henstra A. M. Annan F. J. (2015). Whole genome sequence and manual annotation of Clostridium autoethanogenum, an industrially relevant bacterium. BMC Genomics 16 (1085), 1085. 10.1186/s12864-015-2287-5 26692227 PMC4687164

[B27] Jiang W. Feng S. Huang S. Yu W. Li G. Yang G. (2018). BE-PLUS: A new base editing tool with broadened editing window and enhanced fidelity. Cell Res. 28 (8), 855–861. 10.1038/s41422-018-0052-4 29875396 PMC6082914

[B28] Jinek M. Chylinski K. Fonfara I. Hauer M. Doudna J. A. Charpentier E. (2012). A Programmable Dual-RNA–Guided DNA Endonuclease in adaptive bacterial immunity. Science 337 (6096), 816–821. 10.1126/science.1225829 22745249 PMC6286148

[B29] Kantor A. McClements M. Maclaren R. (2020). Crispr-cas9 dna base-editing and prime-editing. In Int. J. Mol. Sci. 21 (17), 6240. 10.3390/ijms21176240 32872311 PMC7503568

[B30] Komor A. C. Kim Y. B. Packer M. S. Zuris J. A. Liu D. R. (2016). Programmable editing of a target base in genomic DNA without double-stranded DNA cleavage. Nature 533, 420–424. 10.1038/nature17946 27096365 PMC4873371

[B31] Kreuger F. (2012). Trim Galore:: A wrapper tool around Cutadapt and FastQC. Available at: https://www.Bioinformatics.Babraham.Ac.Uk/Projects/Trim_galore/.

[B32] Lee Y. J. Kim S. J. Moon T. S. (2018). Multilevel Regulation of bacterial gene expression with the combined STAR and antisense RNA system. ACS Synth. Biol. 7 (3), 853–865. 10.1021/acssynbio.7b00322 29429328

[B33] Li G. M. (2008). Mechanisms and functions of DNA mismatch repair. Cell Res. 18 (1), 85–98. 10.1038/cr.2007.115 18157157

[B34] Li H. (2013). Aligning sequence reads, clone sequences and assembly contigs with BWA-MEM.

[B35] Li H. Durbin R. (2010). Fast and accurate long-read alignment with Burrows–Wheeler transform. Bioinformatics 26 (5), 589–595. 10.1093/bioinformatics/btp698 20080505 PMC2828108

[B36] Li H. Durbin R. (2009). Fast and accurate short read alignment with Burrows–Wheeler transform. Bioinformatics 25 (14), 1754–1760. 10.1093/bioinformatics/btp324 19451168 PMC2705234

[B37] Li Q. Seys F. M. Minton N. P. Yang J. Jiang Y. Jiang W. (2019). CRISPR–Cas9 D10A nickase-assisted base editing in the solvent producer Clostridium beijerinckii. Biotechnol. Bioeng. 116 (6), 1475–1483. 10.1002/bit.26949 30739328

[B38] Li X. Wang Y. Liu Y. Yang B. Wang X. Wei J. (2018). Base editing with a Cpf1-cytidine deaminase fusion. Nat. Biotechnol. 36 (4), 324–327. 10.1038/nbt.4102 29553573

[B39] Li Z. Deutscher M. P. (2002). RNase E plays an essential role in the maturation of Escherichia coli tRNA precursors. Rna 8 (1), 97–109. 10.1017/S1355838202014929 11871663 PMC1370232

[B40] Li Z. Gong X. Joshi V. H. Li M. (2005). Co-evolution of tRNA 3′ trailer sequences with 3′ processing enzymes in bacteria. RNA 11 (5), 567–577. 10.1261/rna.7287505 15811923 PMC1370745

[B41] Liew F. E. Nogle R. Abdalla T. Rasor B. J. Canter C. Jensen R. O. (2022). Carbon-negative production of acetone and isopropanol by gas fermentation at industrial pilot scale. Nat. Biotechnol. 40 (3), 335–344. 10.1038/s41587-021-01195-w 35190685

[B42] Liu Z. Shan H. Chen S. Chen M. Song Y. Lai L. (2019). Efficient base editing with expanded targeting scope using an engineered Spy-mac Cas9 variant. Cell Discov. 5 (Issue 1), 58. 10.1038/s41421-019-0128-4 31814995 PMC6888851

[B43] Lu H. Zhang Q. Yu S. Wang Y. Kang M. Han S. (2022). Optimization of CRISPR/Cas9-based multiplex base editing in Corynebacterium glutamicum. Shengwu Gongcheng Xuebao/Chinese J. Biotechnol. 38 (2), 780–795. 10.13345/J.CJB.210109 35234398

[B44] Luo Y. Ge M. Wang B. Sun C. Wang J. Dong Y. (2020). CRISPR/Cas9-deaminase enables robust base editing in Rhodobacter sphaeroides 2.4.1. Microb. Cell Factories 19 (1), 93–15. 10.1186/s12934-020-01345-w PMC7183636 32334589

[B45] Mackie G. A. (2013). RNase E: At the interface of bacterial RNA processing and decay. Nat. Rev. Microbiol. 11 (1), 45–57. 10.1038/nrmicro2930 23241849

[B46] Martin M. (2011). Cutadapt removes adapter sequences from high-throughput sequencing reads. EMBnet.J. 17 (1), 10. 10.14806/ej.17.1.200

[B47] Marx V. (2018). Base editing a CRISPR way. Nat. Methods 15 (10), 767–770. 10.1038/s41592-018-0146-4 30275584

[B48] Minagawa A. Takaku H. Takagi M. Nashimoto M. (2004). A novel Endonucleolytic mechanism to generate the CCA 3′ Termini of tRNA molecules in Thermotoga maritima. J. Biol. Chem. 279 (15), 15688–15697. 10.1074/jbc.M313951200 14749326

[B49] Minton N. P. Ehsaan M. Humphreys C. M. Little G. T. Baker J. Henstra A. M. (2016). A roadmap for gene system development in Clostridium. Anaerobe 41, 104–112. 10.1016/j.anaerobe.2016.05.011 27234263 PMC5058259

[B50] Modrich P. (2006). Mechanisms in eukaryotic mismatch repair. J. Biol. Chem. 281 (41), 30305–30309. 10.1074/jbc.R600022200 16905530 PMC2234602

[B51] Molla K. A. Yang Y. (2019). CRISPR/Cas-Mediated base editing: Technical Considerations and practical applications. Trends Biotechnol. 37 (10), 1121–1142. 10.1016/J.TIBTECH.2019.03.008 30995964

[B52] Mörl M. Marchfelder A. (2001). The final cut: The importance of tRNA 3′‐processing. EMBO Rep. 2 (Issue 1), 17–20. 10.1093/embo-reports/kve006 11252717 PMC1083803

[B53] Ng Y. K. Ehsaan M. Philip S. Collery M. M. Janoir C. Collignon A. (2013). Expanding the Repertoire of gene tools for precise Manipulation of the Clostridium difficile genome: Allelic Exchange using pyrE alleles. PLoS ONE 8 (2), e56051. 10.1371/journal.pone.0056051 23405251 PMC3566075

[B54] Nishida K. Arazoe T. Yachie N. Banno S. Kakimoto M. Tabata M. (2016). Targeted nucleotide editing using hybrid prokaryotic and vertebrate adaptive immune systems. Science 353 (6305), aaf8729. 10.1126/science.aaf8729 27492474

[B55] Nishimasu H. Shi X. Ishiguro S. Gao L. Hirano S. Okazaki S. (2018). Engineered CRISPR-Cas9 nuclease with expanded targeting space. Science 361 (6408), 1259–1262. 10.1126/science.aas9129 30166441 PMC6368452

[B56] Ow M. C. Kushner S. R. (2002). Initiation of tRNA maturation by RNase E is essential for cell viability in E. coli. Genes and Dev. 16 (9), 1102–1115. 10.1101/gad.983502 12000793 PMC186257

[B57] Rice P. Longden I. Bleasby A. (2000). Emboss: The European molecular biology open Software suite. Trends Genet. TIG 16 (6), 276–277. 10.1016/S0168-9525(00)02024-2 10827456

[B58] Sekiya T. Contreras R. Takeya T. Khorana H. G. (1979). Total synthesis of a tyrosine suppressor transfer RNA gene. XVII. Transcription, *in vitro*, of the synthetic gene and processing of the primary transcript to transfer RNA. J. Biol. Chem. 254 (13), 5802–5816. 10.1016/s0021-9258(18)50483-x 109442

[B59] Seys F. M. Humphreys C. M. Tomi-Andrino C. Qi L. Millat T. Yang S. (2023). Target-AID off-target detection in C. autoethanogenum. NCBI. Version 1. Bioproject PRJNA956560. 10.3389/fbioe.2023.1211197 PMC10366002 37496853

[B60] Seys F. M. Rowe P. Bolt E. L. Humphreys C. M. Minton N. P. (2020). A gold standard, CRISPR/Cas9-based complementation strategy reliant on 24 nucleotide bookmark sequences. Genes 11 (4), 458. 10.3390/genes11040458 32340238 PMC7230483

[B61] Spampinato C. P. Gomez R. L. Galles C. Lario L. D. (2009). From bacteria to plants: A compendium of mismatch repair assays. Mutat. Research/Reviews Mutat. Res. 682 (2–3), 110–128. 10.1016/j.mrrev.2009.07.001 19622396

[B62] van der Auwera G. O’Connor B. (2020). Genomics in the Cloud: Using Docker, GATK, and WDL in Terra. 1st Edition. Available at: https://www.Oreilly.Com/Library/View/Genomics-in-the/9781491975183/ .

[B63] Wang L. Xue W. Yan L. Li X. Wei J. Chen M. (2017). Enhanced base editing by co-expression of free uracil DNA glycosylase inhibitor. Cell Res. 27 (10), 1289–1292. 10.1038/cr.2017.111 28849781 PMC5630677

[B64] Wang Y. Liu Y. Liu J. Guo Y. Fan L. Ni X. (2018). Macbeth: Multiplex automated Corynebacterium glutamicum base editing method. Metab. Eng. 47, 200–210. 10.1016/j.ymben.2018.02.016 29580925

[B65] Wang Y. Liu Y. Zheng P. Sun J. Wang M. (2020). Microbial base editing: A Powerful Emerging technology for Microbial genome engineering. Trends Biotechnol. 39, 165–180. 10.1016/j.tibtech.2020.06.010 32680590

[B66] Williams J. S. Kunkel T. A. (2014). Ribonucleotides in DNA: Origins, repair and consequences. DNA Repair 19, 27–37. 10.1016/j.dnarep.2014.03.029 24794402 PMC4065383

[B67] Woods C. Humphreys C. M. Rodrigues R. M. Ingle P. Rowe P. Henstra A. M. (2019). A novel conjugal donor strain for improved DNA transfer into Clostridium spp. Anaerobe 59, 184–191. 10.1016/j.anaerobe.2019.06.020 31269456 PMC6866869

[B68] Woods C. Humphreys C. M. Tomi-Andrino C. Henstra A. M. Köpke M. Simpson S. D. (2022). Required gene Set for autotrophic growth of *Clostridium autoethanogenum* . Appl. Environ. Microbiol. 88 (7), e0247921. 10.1128/aem.02479-21 35285680 PMC9004356

[B69] Xie K. Minkenberg B. Yang Y. (2015). Boosting CRISPR/Cas9 multiplex editing capability with the endogenous tRNA-processing system. Proc. Natl. Acad. Sci. 112 (11), 3570–3575. 10.1073/pnas.1420294112 25733849 PMC4371917

[B70] Zhang Y. Wang J. Wang Z. Zhang Y. Shi S. Nielsen J. (2019). A gRNA-tRNA array for CRISPR-Cas9 based rapid multiplexed genome editing in Saccharomyces cerevisiae. Nat. Commun. 10 (1), 1053. 10.1038/s41467-019-09005-3 30837474 PMC6400946

[B71] Zhao C. Zheng X. Qu W. Li G. Li X. Miao Y.-L. (2017). CRISPR-Offinder: A CRISPR guide RNA design and off-target searching tool for user-defined protospacer adjacent motif. Int. J. Biol. Sci. 13 (12), 1470–1478. 10.7150/ijbs.21312 29230095 PMC5723913

[B72] Zhigang W. Smith D. G. Mosbaugh D. W. (1991). Overproduction and characterization of the uracil-DNA glycosylase inhibitor of bacteriophage PBS2. Gene 99 (1), 31–37. 10.1016/0378-1119(91)90030-F 1902430

[B73] Zuker M. (2003). Mfold web server for nucleic acid folding and hybridization prediction. Nucleic Acids Res. 31 (13), 3406–3415. 10.1093/nar/gkg595 12824337 PMC169194

