# Supplementary material for: Base editing enables duplex point mutagenesis in Clostridium autoethanogenum at the price of numerous off-target mutations
Source: Front Bioeng Biotechnol. 2023 Jul 10;11:1211197. doi: 10.3389/fbioe.2023.1211197 (PMC10366002; doi:10.3389/fbioe.2023.1211197)
Supplement: Supplementary file 1 [file DataSheet1.ZIP › Supplementary material/Key sequences, Table S.1-S.6.docx]

Supplementary Material

Base editing enables duplex point mutagenesis in *Clostridium autoethanogenum* at the price of numerous off-target mutations.

Francois M. Seys^1^, Christopher M. Humphreys^1^, Claudio T. Andrino^1,2,3^, Li Qi^4^, Sheng Yang^5^, Nigel P. Minton^1*^

*** Correspondence:** Corresponding Author: nigel.minton@nottingham.ac.uk

# Cloning and assembly

Unless otherwise specified, all DNA parts were purified by gel purification after PCR amplification or digestion.

## Target-AID constructs

The restriction sites of nCas9 were removed from the vector vFS04_pMTL83151_nCas9_trthl. This plasmid was kindly provided to us by Dr. Daphne Groothuis. This vector places nCas9(D10A) under the control of a truncated, non-functional, P_thl_ promoter from *Clostridium acetobutylicum*. Disrupting five restriction sites required amplifying the whole vector in five parts with Q5® DNA polymerase, each part flanked by ~30 bp overlapping regions which each contain one disrupted restriction sites. This was done with the primers oFS17 & oFS18, oFS19 & oFS20, oFS21 & oFS22, oFS23 & oFS24, and oFS26 & oFS27. The five resulting amplicons were assembled using a NEBuilder® HiFi DNA Assembly Kit and cloned into *E. coli* DH5α to form the vector **vFS08_pMTL83151_nCas9_trthl_RSKO** (for “Restriction Site Knock-Out”).

At last, Cau10061II was removed from *lacI* with the help of NEBuilder® HiFi DNA Assembly Cloning Kit. Dr Ryan Hope kindly provided us with the vector vFS06, which encodes Cas9 under the control of Pfacoid [244] and the LacI repressor. vFS06 was digested with MluI, gel extracted, and mixed with oFS64 annealed with oFS76 in a HiFi reaction. This resulted in the vector **vFS20_LacI_RSKO**.

The assembly of **vFS35_TA_PlacIq_LacI_*pyrE*** is long and convoluted. It started with the amplification of vFS05_Pfacoid_CatP with the primers oFS53 & oFS54 and vFS20_LacI_RSKO with oFS55 & oFS56. These amplicons were combined into vFS30_Pfacoid_LacI with a NEBuilder® HiFi DNA Assembly Kit. In parallel, the gblock gFS02 – encoding the sgRNA cassette – was synthesised by Genescript (NJ, USA) and amplified with oFS34 & oFS35 to create the amplicon oFS34-35. Afterwards, vFS30_Pfacoid_LacI, oFS34-35 and pMTL83151_RSKO were all digested (respectively with XbaI & NdeI, XbaI & AscI, and NdeI & AscI) and ligated into a single vector, vFS13. Meanwhile, vFS08_pMTL83151_nCas9_trthl_RSKO was amplified with oFS30 & oFS31 to produce the amplicon oFS30-31, and the vector vFS07_pmCDA1_COOL_Genescript was amplified with primers oFS28 & oFS29. Finally, vFS13 was digested by NdeI & NotI and used as a backbone during the HiFi assembly of oFS28-29 and oFS30-31 to produce vFS31_TA_PlacIq_LacI. Later, an error in the design of gFS02, was corrected by SalI digestion of vFS31_TA_PlacIq_LacI and subsequent HiFi assembly with the annealing product of primers oFS115 & oFS116 to produce vFS35_TA_PlacIq_LacI_*pyrE*.

In order to assemble **vFS36_TA_PfdxE_pyrE**, the P_fdxE_ promoter and its associated riboswitch were amplified out of pMTL-IC111-E [245] with oFS93 & oFS94, while the Target-AID backbone of vFS35_TA_PlacIq_LacI_*pyrE* was amplified with oFS95 & oFS96. Both parts were combined using a NEBuilder® HiFi DNA Assembly Kit in order to constitute vFS36_TA_PfdxE_pyrE, the P_fdxE_.

## Multiplexing constructs

### First round of mutagenesis

**vFS50_TA_msgRNA** was assembled in two steps. The first step resulted from a 6-parts Hifi assembly. The CLAU532A, CLAU534A, and CLAU1794A spacers and the tfdx, tecT1 and tectyrS terminators were added to the sgRNA scaffold by amplifying vFS36_TA_PfdxE_pyrE with oFS121 & oFS137, oFS140 & oFS141, and oFS144 & oFS145 respectively. Then, the Pcpth and Pcpfdx promoters were amplified from the vectors vFS46 and vFS47, kindly provided by Dr. Katalin Kovacs [246], using primers oFS142 & oFS143 and oFS138 & oFS139, respectively. These five parts were assembled with vFS36 previously digested with SalI & AsiSI. Unfortunately, this led to an incomplete, truncated construct (vFS49) with the region in between oFS121 & oFS139 missing. In the second round, oFS121-137 and oFS138-139 were re-amplified from vFS36_TA_PfdxE_pyrE and vFS47. oFS121-137 and oFS138-139 where then digested with SpeI and ligated overnight. The resulting ligation product was amplified with oFS154 & oFS155. After purification, oFS154-155 was digested alongside vFS49 with SalI & AatII, and both parts were finally ligated together to constitute vFS50_TA_msgRNA.

In order to assemble the CRISPR array of **vFS51_TA_mCRISPR**, two rounds of amplifications were needed. First, the primers oFS129 & oFS130 were annealed with each other and amplified with oFS131 & oFS158. Then, the resulting amplicon was itself amplified with oFS159 & oFS160. In parallel, the tracrRNA was also reconstituted in two consecutive rounds of amplifications: first, vFS36_TA_PfdxE_pyrE was amplified with oFS163 & oFS164; then, the resulting amplicon was amplified with oFS165 & oFS166. At last, the Pcpth promoter was extracted from vFS47 with oFS161 & oFS162. vFS36_TA_PfdxE_pyrE was digested with SalI and AsisI, then was combined with oFS159-160, oFS161-162 and oFS165-166 into a HiFi assembly to produce vFS51_TA_mCRISPR.

The assembly of **vFS48_TA_mtRNA** was much simpler. The protospacer and tRNA sequences of each sgRNA were simply added by amplification of vFS36_TA_PfdxE_pyrE with oFS121 & oFS122, oFS123 & oFS124, and oFS125 & oFS126. Meanwhile, vFS36_TA_PfdxE_pyrE was digested with SalI & AsiSI. Afterwards, all parts were used in a HiFi assembly to create vFS48_TA_mtRNA.

**vFS52_TA_CA532A, vFS53_TA_CA534A** and **vFS54_TA_CA1794A** were assembled by standard sgRNA exchange HiFi assembly. vFS36_TA_PfdxE_pyrE was digested with SalI, then used in a HiFi assembly with the annealing products of oFS167 & oFS168, oFS169 & oFS170, or oFS171 & oFS172, respectively.

### Second round of mutagenesis

**vFS57_TA_msgRNA_CA1794B** was assembled through simple sgRNA cassette exchange. The primers oFS195 & oFS196 were annealed together, then used in a HiFi assembly with a SalI-digested vFS50_TA_msgRNA vector. The same method was used to change the sgRNA cassette of vFS54_TA_CA1794A using the primers oFS193 & oFS194 to create the vector **vFS58_TA_CA1794B**.

In order to replace one of the two tRNAs of vFS48_TA_mtRNA and replace the CLAU1794A protospacer, the whole array had to be replaced. Consequently, vFS48_TA_mtRNA was amplified with oFS121 & oFS197 and oFS198 & oFS199, while vFS36_TA_PfdxE_pyrE was being amplified with oFS126 & oFS200. vFS48_TA_mtRNA was digested with SalI & AsiSI, and all four parts were fused in a HiFi assembly, resulting in the vector **vFS56_TA_mTHRtRNA**.

Finally, three different protospacers targeting CLAU_1794 were inserted into vFS57_TA_msgRNA_CA1794B by digesting in with SalI & XhoI, and by amplifying this vector with oFS249 & oFS250 and oFS251 & oFS252. All three parts were combined into a new vector (**vFS74_mTA-NG_CA1794DFG**) during a HiFi assembly.

### Third round of mutagenesis

UGI and its LVA-tag was codon-optimized to by Genscript and synthesized by IDT with 35 bp overhangs (gFS05). vFS57_TA_msgRNA_CA1794B was digested with NotI then re-circularized with gFS05 in a HiFi assembly to constitute the vector **vFS75_mTA-UGILVA**.

Shorter sgRNAs were inserted into vFS57_TA_msgRNA_CA1794B by amplifying it with oFS232 & oFS233, digesting it with AatII & XhoI, and combining both parts in a HiFi assembly. Unfortunately, this resulted in the vector vFS66, which had point mutations in one of its sgRNA scaffolds. To fix this, vFS36 was amplified with oFS145 & oFS284, and vFS66 was digested with XhoI. Both parts were combined in a HiFi assembly, which resulted in the vector **vFS94_mTA_trsgRNA**.

### Fourth round of mutagenesis

In order to assemble **vFS72_mTA-NG**, vFS57_TA_msgRNA_CA1794B was used as template for five PCR amplifications – respectively with primers oFS28 & oFS234, oFS235 & oFS236, oFS237 & oFS238, oFS239 & oFS240, and oFS241 & oFS242. VFS57_TA_msgRNA_CA1794B was then digested with XhoI & NotI, and all six parts were assembled by HiFi assembly.

vFS72_mTA-NG was digested with NotI then re-circularized with gFS05 in a HiFi assembly to constitute the vector vFS79_mTA-NG-UGILVA. This plasmid was then linearized with NotI once again, and the GLVA tag was removed by HiFi with the annealing product of oFS301 & oFS302 to produce the plasnid **vFS103_mTA-NG-UGI_NoLVA**.

## List of primers and oligos

Table S. 1: Sequence, annealing template and association of each primer used in this project. Uppercase letters are used to represent the annealing region of each primer; conversely, when present, lowercase letter represent the overhand of each primer. The purpose of a primer is either to be used for sequencing (Seq) or to assemble a new vector (vFSxx).

| Label | Sequence | Tm (°C) | Annealing template | | Use with | Purpose | | | |  |
| --- | --- | --- | --- | --- | --- | --- | --- | --- | --- | --- |
| oFS017 | gcttaatctatttggccttagcgcaCATGATTAAGTTTCGTGGTC | 58 | vFS04 | | oFS018 | vFS08 | | | |  |
| oFS018 | tccacttgcgtttATAGGGTTTTCTTCAAATAATTG | 55.5 | vFS04 | | oFS017 | vFS08 | | | |  |
| oFS019 | aagaaaaccctatAAACGCAAGTGGAGTAGATG | 62.1 | vFS04 | | oFS020 | vFS08 | | | |  |
| oFS020 | gaggtgagcataaGTTTTAAGTCTTTCCTCAATCATC | 58.8 | vFS04 | | oFS019 | vFS08 | | | |  |
| oFS021 | aaagacttaaaacTTATGCTCACCTCTTTGATG | 59.1 | vFS04 | | oFS022 | vFS08 | | | |  |
| oFS022 | ttacgttgagtgaTTAATTTGGCGTTTAGAAGTTG | 58.4 | vFS04 | | oFS021 | vFS08 | | | |  |
| oFS023 | aacgccaaattaaTCACTCAACGTAAGTTTG | 56 | vFS04 | | oFS024 | vFS08 | | | |  |
| oFS024 | acgcatcatgggcatgatggtaattgttTATCTCACGTACTTTATAGAATTG | 56.4 | vFS04 | | oFS023 | vFS08 | | | |  |
| oFS026 | TGCGCTAAGGCCAAATAGATTAAGC | 67 | vFS04 | | oFS027 | vFS08 | | | |  |
| oFS027 | AACAATTACCATCATGCCCATG | 63 | vFS04 | | oFS026 | vFS08 | | | |  |
| oFS028 | tttatctacaatttttttatcaggaaacagctatgaccgcggccgcTTATCCTGAACCTCTTGAAAC | 58.9 | vFS07 | | oFS029 | vFS31, vFS72 | | | |  |
| oFS029 | gctaggaggtgacAGTAGAGCAGATCCTAAG | 56.2 | vFS07 | | oFS028 | vFS31 | | | |  |
| oFS030 | ttcttaggatctgctctactGTCACCTCCTAGCTGACTC | 59 | vFS04 | | oFS031 | vFS31 | | | |  |
| oFS031 | gattaaaattttaaggaggtgtatttcatATGGATAAGAAATACTCAATAGGC | 59 | vFS04 | | oFS030 | vFS31 | | | |  |
| oFS034 | cgatggtgtcTCTAGATTTATATTTAGTCCCTTGCCTTGCCTAC | 68 | gFS02 | | oFS035 | vFS13 | | | |  |
| oFS035 | tttcaatttggcgcgcctaaaagtaaaGCGATCGCATAAAAATAAGAAGCCTGC | 69 | gFS02 | | oFS034 | vFS13 | | | |  |
| oFS057 | GAAACTTAATCATATGCGCTAAGG | 61 | Cas9 | |  | Seq. | | | |  |
| oFS058 | ATGGATAAGAAATACTCAATAGGCTTAG | 61 | Cas9 | |  | Seq. | | | |  |
| oFS059 | GCTTTGTCATTGGGTTTGAC | 62 | Cas9 | |  | Seq. | | | |  |
| oFS060 | GTCGATAAAGGTGCTTCAGC | 63 | Cas9 | |  | Seq. | | | |  |
| oFS061 | GAACATATTGCAAATTTAGCTGG | 58 | Cas9 | |  | Seq. | | | |  |
| oFS062 | CTGACTTCCGAAAAGATTTCC | 61 | Cas9 | |  | Seq. | | | |  |
| oFS063 | GAGTTAGAAAACGGTCGTAAACG | 63 | Cas9 | |  | Seq. | | | |  |
| oFS064 | CGGCGGTGCACAATCTTCTCGCGCAACGCGTCAGTGGGCTGATCATaAACTATCCGCTGGATGACCAGGATG | N/A | vFS06 | | oFS076 | vFS20 | | | |  |
| oFS076 | GCAATGGCATCCTGGTCATCCAGCGGATAGTTTATGATCAGCCCACTGACGCGTTGCGCGAGAAGATTGTGCACCGCCGCTTTAC | N/A | vFS06 | | oFS064 | vFS20 | | | |  |
| oFS090 | GATTTTTCTCCGTGAGCGTTATG | 64 | *pyrE* | |  | Seq. | | | |  |
| oFS091 | gcaATTGTTCCACAAAGTTTCCTTAAAGACG | 65 | vFS19 | | oFS092 | vFS39 | | | |  |
| oFS092 | ATCGACATCATAATCACTTAAACGATTAATATC | 62 | vFS19 | | oFS091 | vFS39 | | | |  |
| oFS093 | CTTGTTGTTACCTCCTTAGCAG | 63 | pMTL-IC111-E | oFS094 | | | vFS36 | | | |
| oFS094 | GTGTAGTAGCCTGTGAAATAAGTAAG | 62 | pMTL-IC111-E | oFS093 | | | vFS36 | | | |
| oFS095 | gcagcaccctgctaaggaggtaacaacaagATGGATAAGAAATACTCAATAGGCTTAG | 61 | vFS35 | | oFS096 | vFS36 | | | |  |
| oFS096 | ccttacttatttcacaggctactacacTCTAGATTTATATTTAGTCCCTTGCC | 61 | vFS35 | | oFS095 | vFS36 | | | |  |
| oFS105 | gagcttatgcaattcaagtaggtactgcaaac | 59 | *pyrE* | | oFS106 | Seq. | | | |  |
| oFS106 | catcaaagctatactattttccgtatttacatttggg | 57 | *pyrE* | | oFS105 | Seq. | | | |  |
| oFS115 | ACATATATAAATCTTAAGGAGGAGTTTTCGTCGACaattcaggaattaggtggagGTTTTAGAGCTAGAAATAGCAAGTTAAAAT | N/A | oFS116 | oFS116 | | | vFS35 | |  |  |
| oFS116 | ATTTTAACTTGCTATTTCTAGCTCTAAAACctccacctaattcctgaattGTCGACGAAAACTCCTCCTTAAGATTTATATATGT | N/A | oFS115 | oFS115 | | | vFS35 | |  |  |
| oFS121 | tttccacatatataaatcttaaggaggagttttcgtcgacctccagtcaggtgttgtgcagttttagagctagaaatagcAAGTTAAAATAAGGCTAGTCCGTTATC | 62 | vFS36 | | oFS122 | vFS49, vFS48, vFS56 | | | |  |
| oFS122 | ggaattcctccaaaattggtgctggcaataggaattgaacccataacctactgattacaagtcagttgctctaccaattgagctatgccagcAAAAAAAGCACCGACTCG | 60 | vFS36 | | oFS121 | vFS48 | | | |  |
| oFS123 | caattcctattgccagcaccaattttggaggaattcccgagtagcccaatgtctagctgggagttttagagctagaaatagcAAGTTAAAATAAGGCTAGTCCGTTATC | 62 | vFS36 | | oFS124 | vFS48 | | | |  |
| oFS124 | gctattccgccaaatctggttgcgggggtaggacttgaacctacgaccttcgggttatgagcccgacgagctgccagctgctccaccccgcgAAAAAAAGCACCGACTCG | 60 | vFS36 | | oFS123 | vFS48 | | | |  |
| oFS125 | caagtcctacccccgcaaccagatttggcggaatagctcagctaaacaagcaattgttccgttgttttagagctagaaatagcAAGTTAAAATAAGGCTAGTCCGTTATC | 62 | vFS36 | | oFS126 | vFS48 | | | |  |
| oFS126 | caaaaaaataatggcggcgcgcctaaaagtaaagcgatcgcataaaaataagaagcctgcaaatgcaggcttcttatttttatAAAAAAAGCACCGACTCG | 60 | vFS36 | | oFS125 | vFS48, vFS56 | | | |  |
| oFS129 | CCCAAAACtgaaCTCGAGagcccaatgtctagctgggatGTTTTAGAGCTATGCTGTTTTGAATGGTCCCAAAACactaACTAGTaaacaag | 81 | oFS130 | | oFS130 | vFS51 | | | |  |
| oFS130 | cttgtttACTAGTtagtGTTTTGGGACCATTCAAAACAGCATAGCTCTAAAACatcccagctagacattgggctCTCGAGttcaGTTTTGGG | 81 | oFS129 | | oFS129 | vFS51 | | | |  |
| oFS131 | gcaagacgtcctccagtcaggtgttgtgcagttttagagctatgctgttttgaatggtcCCAAAACTGAACTCGAGAG | 60 | oFS129 | oFS158 | | | vFS51 | |  |  |
| oFS137 | gtataaaaataagaagcctgcaaatgcaggcttcttatttttatAAAAAAAGCACCGACTC | 62 | vFS36 | | oFS121 | vFS49 | | | |  |
| oFS138 | gaagcctgcatttgcaggcttcttatttttatactagtGACTTTGTTAAAAAAGTTTAATAAATATAATTTGAATAAATGGTATAAATAGACAGATATTTAG | 65 | vFS47 | | oFS139 | vFS49 | | | |  |
| oFS139 | ctcccagctagacattgggctgacgtccaaaaatctatattttttCTAACTGATTTAATTATAACCATAAATTTACTAGC | 64 | vFS47 | | oFS138 | vFS49 | | | |  |
| oFS140 | gaaaaaatatagatttttggacgtcagcccaatgtctagctgggagttttagagctagaaatagcAAGTTAAAATAAGGCTAGTCCGTTATC | 62 | vFS36 | | oFS141 | vFS49 | | | |  |
| oFS141 | caaacaacagataaaacgaaaggcccagtctttcgactgagcctttcgttttatttgatgcctggAAAAAAAGCACCGACTCG | 60 | vFS36 | | oFS140 | vFS49 | | | |  |
| oFS142 | ggctcagtcgaaagactgggcctttcgttttatctgttgtttgtcggtgaacgctctcagatctaattaaatttttaatACGGTATAGGGGTATTCTTTAGC | 63 | Ppffdx | | oFS143 | vFS49 | | | |  |
| oFS143 | caacggaacaattgcttgtttctcgagTACCATATTTATATTATCATATTTTTGCTAATTTTTAAAGTATTTAATATCTC | 62 | Ppffdx | | oFS142 | vFS49 | | | |  |
| oFS144 | gataatataaatatggtactcgagaaacaagcaattgttccgttgttttagagctagaaatagcAAGTTAAAATAAGGCTAGTCCGTTATC | 62 | vFS36 | | oFS145 | vFS49 | | | |  |
| oFS145 | caaaaaaataatggcggcgcgcctaaaagtaaagcgatcgcaaataaaaaacgccccttcgtttacacgaagggacgattgattatAAAAAAAGCACCGACTC | 60 | vFS36 | | oFS144, oFS284 | vFS49, vFS94 | | | |  |
| oFS154 | tttccacatatataaatcttaaggagg | 59 | oFS121-oFS139 | oFS155 | | | vFS50 | | | |
| oFS155 | ctcccagctagacattg | 59 | oFS121-oFS139 | oFS154 | | | vFS50 | | | |
| oFS158 | ctaaaacaacggaacaattGCTTGTTTACTAGTTAGTGTTTTGGGAC | 65 | oFS130 | oFS131 | | | Seq. | | | |
| oFS159 | gatttccacatatataaatcttaaggaggagttttcgtcgacgttttagagctatgctgttttgaatggtcccaaaacgcaagacgtcCTCCAGTCAGGTGTTGTGCAG | 68 | oFS131-oFS158 | | oFS160 | vFS51 | | | |  |
| oFS160 | agtcagatctataaaaataagaagcctgcaaatgcaggcttcttatttttatgttttgggaccattcaaaacagcatagctCTAAAACAACGGAACAATTGCTTGTTTAC | 65 | oFS131-oFS158 | | oFS159 | vFS51 | | | |  |
| oFS161 | catttgcaggcttcttatttttatagatctGACTTTGTTAAAAAAGTTTAATAAATATAATTTGAATAAATGGTATAAATAGACAGATATTTAG | 65 | vFS47 | | oFS162 | vFS51 | | | |  |
| oFS162 | CAAAAATCTATATTTTTTCTAACTGATTTAATTATAACCATAAATTTACTAGC | 64 | vFS47 | | oFS161 | vFS51 | | | |  |
| oFS163 | aaatatagatttttgggaaccattcaaaacagcATAGCAAGTTAAAATAAGGCTAGTCCG | 64 | vFS36 | | oFS164 | vFS51 | | | |  |
| oFS164 | gagagcgttcaccgacaaacaacagataaaacgaaaggcccagtctttcgactgagcctttcgttttatttgatgcctggAAAAAAAGCACCGACTCGGTG | 66 | vFS36 | | oFS163 | vFS51 | | | |  |
| oFS165 | tggttataattaaatcagttagaaaaaatataGATTTTTGGGAACCATTCAAAACAGC | 65 | oFS163-oFS164 | oFS166 | | | vFS51 | |  |  |
| oFS166 | aaaaaaataatggcggcgcgcctaaaagtaaagcgatcgcGAGAGCGTTCACCGACAAAC | 66 | oFS163-oFS164 | oFS165 | | | vFS51 | |  |  |
| oFS167 | ATCTTAAGGAGGAGTTTTCGTCGACctccagtcaggtgttgtgcaGTTTTAGAGCTAGAAATAGCAAGTT | N/A | vFS36 | | oFS168 | vFS52 | | | |  |
| oFS168 | AACTTGCTATTTCTAGCTCTAAAACtgcacaacacctgactggagGTCGACGAAAACTCCTCCTTAAGAT | N/A | vFS36 | | oFS167 | vFS52 | | | |  |
| oFS169 | ATCTTAAGGAGGAGTTTTCGTCGACagcccaatgtctagctgggaGTTTTAGAGCTAGAAATAGCAAGTT | N/A | vFS36 | | oFS170 | vFS53 | | | |  |
| oFS170 | AACTTGCTATTTCTAGCTCTAAAACtcccagctagacattgggctGTCGACGAAAACTCCTCCTTAAGAT | N/A | vFS36 | | oFS169 | vFS53 | | | |  |
| oFS171 | ATCTTAAGGAGGAGTTTTCGTCGACaaacaagcaattgttccgttGTTTTAGAGCTAGAAATAGCAAGTT | N/A | vFS36 | | oFS171 | vFS54 | | | |  |
| oFS172 | AACTTGCTATTTCTAGCTCTAAAACaacggaacaattgcttgtttGTCGACGAAAACTCCTCCTTAAGAT | N/A | vFS36 | | oFS170 | vFS54 | | | |  |
| oFS173 | CATACTGGCACAACATTTGC | 62 | CLAU532 | oFS174 | | | Seq. | | | |
| oFS174 | TCACATTTTTTTAATGCGACAG | 59 | CLAU532 | oFS173 | | | Seq. | | | |
| oFS175 | CCCCAATAGTTAAATTCTAAAAAAGTAATG | 60 | CLAU534 | oFS176 | | | Seq. | | | |
| oFS176 | GAGCATACTGCTTTAGTTTG | 58 | CLAU534 | oFS175 | | | Seq. | | | |
| oFS177 | GTTTATATTAGGGTTATATTGAGGTC | 58 | CLAU1794 | oFS178 | | | Seq. | | | |
| oFS178 | GTTGATTGGTACGAATTTTGC | 60 | CLAU1794 | oFS177 | | | | Seq. | | |
| oFS193 | ATCTTAAGGAGGAGTTTTCGTCGACatcacaatgtttagcaggtaGTTTTAGAGCTAGAAATAGCAAGTT | N/A | vFS54 | | oFS194 | vFS58 | | | |  |
| oFS194 | AACTTGCTATTTCTAGCTCTAAAACtacctgctaaacattgtgatGTCGACGAAAACTCCTCCTTAAGAT | N/A | vFS54 | | oFS193 | vFS58 | | | |  |
| oFS195 | TAGCAAAAATATGATAATATAAATATGGTACTCGAGatcacaatgtttagcaggtaGTTTTAGAGCTAGAAATAGCAAGTT | N/A | vFS50 | | oFS196 | vFS57 | | | |  |
| oFS196 | AACTTGCTATTTCTAGCTCTAAAACtacctgctaaacattgtgatCTCGAGTACCATATTTATATTATCATATTTTTGCTA | N/A | vFS50 | | oFS195 | vFS57 | | | |  |
| oFS197 | ctaaaacTCCCAGCTAGACATTGGG | 64 | vFS48 | | oFS121 | vFS56 | | | |  |
| oFS198 | cccgagtagcccaatgtctagctgggagttttagagctagaaatagcAAGTTAAAATAAGGCTAGTCCGTTATC | 62 | vFS48 | | oFS199 | vFS56 | | | |  |
| oFS199 | ctaaaactacctgctaaacattgtgatACTCGGGAATTCCTCC | 60 | vFS48 | | oFS198 | vFS56 | | | |  |
| oFS200 | cccgagtatcacaatgtttagcaggtagttttagagctagaaatagcAAGTTAAAATAAGGCTAGTCCGTTATC | 62 | vFS36 | | oFS126 | vFS56 | | | |  |
| oFS209 | GGAGATCTTTAGAAGTCCAAG | 59 | CLAU532 | |  | Seq. | | | |  |
| oFS210 | TGTTACTCATGTATTTGATGGTG | 60 | CLAU532 | |  | Seq. | | | |  |
| oFS211 | TGCTGCTAAAGCAATGTG | 60 | CLAU534 | |  | Seq. | | | |  |
| oFS212 | TTTATAGCTCACAATGCCATG | 60 | CLAU534 | |  | Seq. | | | |  |
| oFS213 | ACCTCGAAGCCTTAACTG | 61 | CLAU1794 | |  | Seq. | | | |  |
| oFS214 | CATTAACTCATGCACTAGAAGC | 61 | CLAU1794 | |  | Seq. | | | |  |
| oFS215 | AATCAATGCACGATGCAG | 60 | pyrE LHA | |  | Seq. | | | |  |
| oFS216 | AGTCTAAGGATGCAGCAAG | 62 | pyrE RHA | |  | Seq. | | | |  |
| oFS232 | cagttagaaaaaatatagatttttggacgtccccaatgtctagctgggagttttagagctagaaatagcAAGTTAAAATAAGGCTAGTCCGTTATC | 62 | vFS57 | | oFS233 | vFS66 | | | |  |
| oFS233 | cttattttaacttgctatttctagctctaaaactacctgctaaacattgtgCTCGAGTACCATATTTATATTATCATATTTTTGCTAATTTTTAAAGTATTTAATATCTC | 62 | vFS57 | | oFS232 | vFS66 | | | |  |
| oFS234 | cagagcttttaaatattttgatacaacaattgatcgtaaagtatatagaagtACAAAAGAAGTTTTAGATGCC | 60 | vFS57 | | oFS28 | vFS72 | | | |  |
| oFS235 | cgatcaattgttgtatcaaaatatttaaaagctctGGGAGCTCCAAGATTCGTCAACGTAAATAAATG | 62 | vFS57 | | oFS236 | vFS72 | | | |  |
| oFS236 | cggtcgtaaacggatgctggctagtgccagatttTTACAAAAAGGAAATGAGCTGGC | 63 | vFS57 | | oFS235 | vFS72 | | | |  |
| oFS237 | aaatctggcactagccAGCATCCGTTTACGACCGTTTTC | 65 | vFS57 | | oFS238 | vFS72 | | | |  |
| oFS238 | caaggagtcaattagaccaaaaagaaattcggacaagcttattgctcgtaaaaaagactgggatccaaaaaaatatggtggttttgtaAGTCCAACGGTAGCTTATTCAG | 64 | vFS57 | | oFS237 | vFS72 | | | |  |
| oFS239 | cgagcaataagcttgtccgaatttctttttggtctAATTGACTCCTTGGAGAATCCGCCTGTCTGTACTTCTGTTTTCTTG | 63 | vFS57 | | oFS240 | vFS72 | | | |  |
| oFS240 | acaaattgtgccttctgcctgacgtcCAAAAATCTATATTTTTTCTAACTGATTTAATTATAACCATAAATTTACTAGCTTTC | 66 | vFS57 | | oFS239 | vFS72 | | | |  |
| oFS241 | gatttttggacgtcaggcagaaggcacaatttgtgttttagagctagaaatagcAAGTTAAAATAAGGCTAGTCCGTTATC | 62 | vFS57 | | oFS242 | vFS72 | | | |  |
| oFS242 | tattttaacttgctatttctagctctaaaacgaatttgggccagattggatctcgagTACCATATTTATATTATCATATTTTTGCTAATTTTTAAAGTATTTAATATCTC | 62 | vFS57 | | oFS241 | vFS72 | | | |  |
| oFS249 | gatttccacatatataaatcttaaggaggagttttcgtcgacagacaaaaagctaaatttgtgttttagagctagaaatagcAAGTTAAAATAAGGCTAGTCCGTTATC | 62 | vFS57 | | oFS250 | vFS74 | | | |  |
| oFS250 | catacctgctaaacattgtgagacgtcCAAAAATCTATATTTTTTCTAACTGATTTAATTATAACCATAAATTTACTAGCTTTC | 62 | vFS57 | | oFS249 | vFS74 | | | |  |
| oFS251 | gatttttggacgtctcacaatgtttagcaggtatgttttagagctagaaatagcAAGTTAAAATAAGGCTAGTCCGTTATC | 62 | vFS57 | | oFS252 | vFS74 | | | |  |
| oFS252 | tattttaacttgctatttctagctctaaaactaaaacaggagctgtatggcctcgagTACCATATTTATATTATCATATTTTTGCTAATTTTTAAAGTATTTAATATCTC | 62 | vFS57 | | oFS251 | vFS74 | | | |  |
| oFS278 | ctaataatttaattgtcaattctgcatcgtg | 62 | *pyrE* | | oFS105 | Seq. | | | |  |
| oFS279 | gcagacaaattagagaatgttgactatg | 63 | *pyrE* | |  | Seq. | | | |  |
| oFS284 | gataatataaatatggtaCTCGAGcacaatgtttagcaggtagttttagagctagaaatagcAAGTTAAAATAAGGCTAGTCCGTTATC | 60 | vFS36 | | oFS145 | vFS94 | | | |  |
| oFS301 | caatttttttatcaggaaacagctatgaccgcggccgcTTATAACATTTTTATTTTATTTTCTCCATTAC |  | oFS302 | | oFS302 | vFS103 | | | |  |
| oFS302 | GTAATGGAGAAAATAAAATAAAAATGTTATAAgcggccgcggtcatagctgtttcctgataaaaaaattg |  | oFS301 | | oFS301 | vFS103 | | | |  |
| oFS309 | GCGATTGTACATCCTCTAGC | 62 | CLAU532 |  | | | Seq. | | | |
| oFS310 | TCTGCACCATCAAATACATGAG | 62 | CLAU532 |  | | | Seq. | | | |
| oFS313 | cactaacaatatactctgagacttatcatc | 62 | *pyrE* |  | | | Seq. | | | |
| oFS314 | gaaataagaggaataatttaggaggac | 60 | *pyrE* |  | | | Seq. | | | |

## List of vectors

Table S. 2: Summary of all vectors used in this project. For each vector, its name, usage and source are presented. All vectors use *catP* (chloramphenicol resistance gene), except the ones followed by *(Ampicilin resistance).

| Label | Usage | Source |
| --- | --- | --- |
| vFS04_ pMTL83151_nCas9_trthl | Amplify spCas9n | Daphne Groothuis |
| vFS05_ Pfacoid_CatP | Amplify Pfacoid | Hengzeng |
| vFS06_FacOID_cas9_placIQ_lacI | Amplify PlacIq-LacI | Ryan Hope |
| vFS07_ pmCDA1_COOL_Genescript* | Amplify pMCDA1 to make the AID of Target-AID | This study |
| vFS08_pMTL83151_nCas9_trthl_RSKO | Amplify spCas9n_RSKO | This study |
| vFS13_vFS30_vFS45_col3 | Host vector for Target-AID protein | This study |
| vFS20_ LacI_RSKO | Amplifying a *lacI* without *C. autoethanogenum* restriction site. | This study |
| vFS30_ Pfacoid_LacIq | Template for Target-AID assembly | This study |
| vFS31_vFS13_vFS08_vFS07_col13 | Final Target-AID construct targeting *bdh* in *C. autoethanogenum* | This study |
| vFS32_ pMTL-IC111-E | Amplify rb3 repression system to replace LacIq in Target-AID. | (Cañadas, 2019) |
| vFS35_TA_Placiq_LacI_*pyrE* | Test Pfacoid-LacI repression system with Target-AID. | This study |
| vFS36_ TA_P_fdxE__*pyrE* | Delete *pyrE* using Target-AID | This study |
| vFS46_pMTL8225x_TT_Cpf_fdx_catP_CACthlRBS | Amplify Ppffdx with oFS142-143 | (Pander, 2017) |
| vFS47_pMTL8225x_TT_Cpf_thl_CACthlRBS_catP | Amplify Ppfthl with oFS138-139 | (Pander, 2017) |
| vFS48_ TA_mtRNA | Knock out CLAU532,534,1794 in one go; test tRNA as a multiplexing tool | This study |
| vFS49_Truncated_ TA_msgRNA | Knock out CLAU532,534,1794 in one go; Truncated, do not use | This study |
| vFS50_TA_msgRNA | Knock out CLAU532,534,1794 in one go; | This study |
| vFS51_TA_mCRISPR | Knock out CLAU532,534,1794 in one go; test CRISPR DR and traRNA as a multiplexing tool | This study |
| vFS52_ TA_CA532A | Knock out CLAU532; control with single target for multiplexing experiment. | This study |
| vFS53_ TA_CA534A | Knock out CLAU534; control with single target for multiplexing experiment. | This study |
| vFS54_ TA_CA1794A | Knock out CLAU1794; control with single target for multiplexing experiment. | This study |
| vFS56_mTHRtRNA | Troubleshoot vFS48 with alternative CLAU1794sgRNA and same tRNA twice in the array | This study |
| vFS57_ TA_msgRNA_CA1794B | Troubleshoot vFS50 with alternative CLAU1794sgRNA | This study |
| vFS58_TA_CA1794B | Knock out CLAU1794; control with single target for multiplexing experiment. | This study |
| vFS66_TA-msgRNA_short_truncated | Intermediary for the construction of vFS94_mTA_trsgRNA | This study |
| vFS72_mTA-NG | Knock out CLAU532,534,1794 in one go; test Cas9-NG. | This study |
| vFS74_ mTA-NG_CA1794DFG | Test three additional gRNAs for CLAU1794. | This study |
| vFS75_mTA-UGILVA | Knock out CLAU532,534,1794 in one go; test UGI with GLVA degradation tag. | This study |
| vFS79_mTA-NG-UGILVA | Intermediary for the construction of vFS103_mTA-NG-UGI_NoLVA | This study |
| vFS94_mTA_trsgRNA | Knock out CLAU532,534,1794 in one go; test truncated sgRNA to target cytosines in position -16 from the PAM. | This study |
| vFS103_mTA-NG-UGI_NoLVA | Knock out CLAU532,534,1794 in one go; test UGI fusion withGLVA degradation tag. | This study |

# Key sequences

## Target-AID

### PfdxE

gtgtagtagcctgtgaaataagtaaggaaaaaaaagaagtaagtgttatatatgatgattattttgtagatgtagataggataatagaatccatagaaaatataggttatacagttatataaaaattactttaaaaattaataaaaacatggtaaaatataaatcggtaccaatacgactcactataggttccggtgataccagcatcgtcttgatgcccttggcagcaccctgctaaggaggtaacaacaag

### nCas9(D10A)-RSKO

ATGGATAAGAAATACTCAATAGGCTTAGcTATCGGCACAAATAGCGTCGGATGGGCGGTGATCACTGATGAATATAAGGTTCCGTCTAAAAAGTTCAAGGTTCTGGGAAATACAGACCGCCACAGTATCAAAAAAAATCTTATAGGGGCTCTTTTATTTGACAGTGGAGAGACAGCGGAAGCGACTCGTCTCAAACGGACAGCTCGTAGAAGGTATACACGTCGGAAGAATCGTATTTGTTATCTACAGGAGATTTTTTCAAATGAGATGGCGAAAGTAGATGATAGTTTCTTTCATCGACTTGAAGAGTCTTTTTTGGTGGAAGAAGACAAGAAGCATGAACGTCATCCTATTTTTGGAAATATAGTAGATGAAGTTGCTTATCATGAGAAATATCCAACTATCTATCATCTGCGAAAAAAATTGGTAGATTCTACTGATAAAGCGGATTTGCGCTTAATCTATTTGGCCTTAGCGCAcATGATTAAGTTTCGTGGTCATTTTTTGATTGAGGGAGATTTAAATCCTGATAATAGTGATGTGGACAAACTATTTATCCAGTTGGTACAAACCTACAATCAATTATTTGAAGAAAACCCTATaAACGCAAGTGGAGTAGATGCTAAAGCGATTCTTTCTGCACGATTGAGTAAATCAAGACGATTAGAAAATCTCATTGCTCAGCTCCCCGGTGAGAAGAAAAATGGCTTATTTGGGAATCTCATTGCTTTGTCATTGGGTTTGACCCCTAATTTTAAATCAAATTTTGATTTGGCAGAAGATGCTAAATTACAGCTTTCAAAAGATACTTACGATGATGATTTAGATAATTTATTGGCGCAAATTGGAGATCAATATGCTGATTTGTTTTTGGCAGCTAAGAATTTATCAGATGCTATTTTACTTTCAGATATCCTAAGAGTAAATACTGAAATAACTAAGGCTCCCCTATCAGCTTCAATGATTAAACGCTACGATGAACATCATCAAGACTTGACTCTTTTAAAAGCTTTAGTTCGACAACAACTTCCAGAAAAGTATAAAGAAATCTTTTTTGATCAATCAAAAAACGGATATGCAGGTTATATTGATGGGGGAGCTAGCCAAGAAGAATTTTATAAATTTATCAAACCAATTTTAGAAAAAATGGATGGTACTGAGGAATTATTGGTGAAACTAAATCGTGAAGATTTGCTGCGCAAGCAACGGACCTTTGACAACGGCTCTATTCCCCATCAAATTCACTTGGGTGAGCTGCATGCTATTTTGAGAAGACAAGAAGACTTTTATCCATTTTTAAAAGACAATCGTGAGAAGATTGAAAAAATCTTGACTTTTCGAATTCCTTATTATGTTGGTCCATTGGCGCGTGGCAATAGTCGTTTTGCATGGATGACTCGGAAGTCTGAAGAAACAATTACCCCATGGAATTTTGAAGAAGTTGTCGATAAAGGTGCTTCAGCTCAATCATTTATTGAACGCATGACAAACTTTGATAAAAATCTTCCAAATGAAAAAGTACTACCAAAACATAGTTTGCTTTATGAGTATTTTACGGTTTATAACGAATTGACAAAGGTCAAATATGTTACTGAAGGAATGCGAAAACCAGCATTTCTTTCAGGTGAACAGAAGAAAGCCATTGTTGATTTACTCTTCAAAACAAATCGAAAAGTAACCGTTAAGCAATTAAAAGAAGATTATTTCAAAAAAATAGAATGTTTTGATAGTGTTGAAATTTCAGGAGTTGAAGATAGATTTAATGCTTCATTAGGTACCTACCATGATTTGCTAAAAATTATTAAAGATAAAGATTTTTTGGATAATGAAGAAAATGAAGATATCTTAGAGGATATTGTTTTAACATTGACCTTATTTGAAGATAGGGAGATGATTGAGGAAAGACTTAAAACtTATGCTCACCTCTTTGATGATAAGGTGATGAAACAGCTTAAACGTCGCCGTTATACTGGTTGGGGACGTTTGTCTCGAAAATTGATTAATGGTATTAGGGATAAGCAATCTGGCAAAACAATATTAGATTTTTTGAAATCAGATGGTTTTGCCAATCGCAATTTTATGCAGCTGATCCATGATGATAGTTTGACATTTAAAGAAGACATTCAAAAAGCACAAGTGTCTGGACAAGGCGATAGTTTACATGAACATATTGCAAATTTAGCTGGTAGCCCTGCTATTAAAAAAGGTATTTTACAGACTGTAAAAGTTGTTGATGAATTGGTCAAAGTAATGGGGCGGCATAAGCCAGAAAATATCGTTATTGAAATGGCACGTGAAAATCAGACAACTCAAAAGGGCCAGAAAAATTCGCGAGAGCGTATGAAACGAATCGAAGAAGGTATCAAAGAATTAGGAAGTCAGATTCTTAAAGAGCATCCTGTTGAAAATACTCAATTGCAAAATGAAAAGCTCTATCTCTATTATCTCCAAAATGGAAGAGACATGTATGTGGACCAAGAATTAGATATTAATCGTTTAAGTGATTATGATGTCGATCACATTGTTCCACAAAGTTTCCTTAAAGACGATTCAATAGACAATAAGGTCTTAACGCGTTCTGATAAAAATCGTGGTAAATCGGATAACGTTCCAAGTGAAGAAGTAGTCAAAAAGATGAAAAACTATTGGAGACAACTTCTAAACGCCAAaTTAATCACTCAACGTAAGTTTGATAATTTAACGAAAGCTGAACGTGGAGGTTTGAGTGAACTTGATAAAGCTGGTTTTATCAAACGCCAATTGGTTGAAACTCGCCAAATCACTAAGCATGTGGCACAAATTTTGGATAGTCGCATGAATACTAAATACGATGAAAATGATAAACTTATTCGAGAGGTTAAAGTGATTACCTTAAAATCTAAATTAGTTTCTGACTTCCGAAAAGATTTCCAATTCTATAAAGTACGTGAGATaAACAATTACCATCATGCCCATGATGCGTATCTAAATGCCGTCGTTGGAACTGCTTTGATTAAGAAATATCCAAAACTTGAATCGGAGTTTGTCTATGGTGATTATAAAGTTTATGATGTTCGTAAAATGATTGCTAAGTCTGAGCAAGAAATAGGCAAAGCAACCGCAAAATATTTCTTTTACTCTAATATCATGAACTTCTTCAAAACAGAAATTACACTTGCAAATGGAGAGATTCGCAAACGCCCTCTAATCGAAACTAATGGGGAAACTGGAGAAATTGTCTGGGATAAAGGGCGAGATTTTGCCACAGTGCGCAAAGTATTGTCCATGCCCCAAGTCAATATTGTCAAGAAAACAGAAGTACAGACAGGCGGATTCTCCAAGGAGTCAATTTTACCAAAAAGAAATTCGGACAAGCTTATTGCTCGTAAAAAAGACTGGGATCCAAAAAAATATGGTGGTTTTGATAGTCCAACGGTAGCTTATTCAGTCCTAGTGGTTGCTAAGGTGGAAAAAGGGAAATCGAAGAAGTTAAAATCCGTTAAAGAGTTACTAGGGATCACAATTATGGAAAGAAGTTCCTTTGAAAAAAATCCGATTGACTTTTTAGAAGCTAAAGGATATAAGGAAGTTAAAAAAGACTTAATCATTAAACTACCTAAATATAGTCTTTTTGAGTTAGAAAACGGTCGTAAACGGATGCTGGCTAGTGCCGGAGAATTACAAAAAGGAAATGAGCTGGCTCTGCCAAGCAAATATGTGAATTTTTTATATTTAGCTAGTCATTATGAAAAGTTGAAGGGTAGTCCAGAAGATAACGAACAAAAACAATTGTTTGTGGAGCAGCATAAGCATTATTTAGATGAGATTATTGAGCAAATCAGTGAATTTTCTAAGCGTGTTATTTTAGCAGATGCCAATTTAGATAAAGTTCTTAGTGCATATAACAAACATAGAGACAAACCAATACGTGAACAAGCAGAAAATATTATTCATTTATTTACGTTGACGAATCTTGGAGCTCCCGCTGCTTTTAAATATTTTGATACAACAATTGATCGTAAACGATATACGTCTACAAAAGAAGTTTTAGATGCCACTCTTATCCATCAATCCATCACTGGTCTTTATGAAACACGCATTGATTTGAGTCAGCTAGGAGGTGAC

Purpose of SNPs compared to WT: D10A (inactivate RuvC); Disruption of Cau10061II; disruption of NdeI.

### Codon-optimized PmCDA1

agtagagcagatcctaagaagaaaagaaaagttggtggtggtggaagtggtggtggtggaagtgcagaatatgttagagcattatttgattttaatggaaatgatgaagaagatttaccttttaagaaaggagatatacttagaataagagataaaccagaagaacaatggtggaatgcagaagattctgaaggaaaaagaggaatgatacctgtaccttatgtagaaaaatattcaggagattataaagatcatgatggtgactacaaagatcatgatatagattataaagatgatgatgataaaagtagaATGACTGATGCTGAATATGTTAGAATACATGAAAAATTAGATATATATACATTTAAAAAACAATTTTTCAATAATAAAAAATCTGTATCACATAGATGTTATGTATTATTTGAATTAAAAAGAAGAGGAGAAAGAAGAGCATGTTTTTGGGGATATGCTGTAAATAAACCTCAATCAGGAACTGAAAGAGGAATACATGCAGAAATATTTAGTATAAGAAAAGTAGAAGAATATTTAAGAGATAATCCTGGACAATTTACAATAAACTGGTATAGTTCTTGGAGTCCATGTGCAGATTGTGCTGAAAAAATATTAGAATGGTATAATCAAGAACTTAGAGGAAATGGACATACTCTTAAAATATGGGCATGTAAACTTTATTATGAAAAGAATGCTAGAAATCAAATAGGATTATGGAATTTAAGAGATAATGGAGTAGGATTAAATGTAATGGTATCTGAACATTATCAATGTTGTAGAAAAATATTTATACAATCAAGTCATAATCAATTAAATGAAAATAGATGGCTTGAAAAAACACTTAAAAGAGCTGAAAAGAGAAGAAGTGAATTATCTATAATGATTCAGGTAAAAATACTTCATACAACAAAATCACCAGCAGTTTCAAGAGGTTCAGGATAA

The linker is in lowercase, the CDS of PmCDA1 is in uppercase.

Linker features: SV40 NLS; GSG linker; SH3 domain; 3xFLAG

### gFS05_UGI-LVA

CTTCATACAACAAAATCACCAGCAGTTTCAAGAATGACAAATTTATCAGATATTATAGAAAAAGAAACAGGAAAACAGTTAGTTATACAAGAATCAATACTTATGTTACCAGAAGAAGTTGAAGAAGTAATAGGAAATAAACCTGAAAGTGATATATTAGTACATACTGCTTATGATGAAAGTACAGATGAAAATGTAATGTTACTTACTTCTGATGCACCTGAATATAAACCTTGGGCTTTAGTAATACAGGATAGTAATGGAGAAAATAAAATAAAAATGTTAGGTCTTGTTGCATAAgcggccgcggtcatagctgtttcctgataaaaaaattgtagataaa

Feature: UGI GLVA degradation tag Overlap region with NotI-linearized vFS72_mTA-NG

## Multiplexing

### msgRNA array

Table S.3: Key parts of the msgRNA array construct. P=promoter, T=terminator, chRNA=chimeric RNA (directly downstream of 20 nt protospacer). Only ParaE still has an RBS.

| **Name** | **Sequence** |
| --- | --- |
| **ParaE** | tttatatttagtcccttgccttgcctacaagggatttcctattcctttcatttacaattcatacgtataaaatccaaatttttcttgacatttatacacataaatattatgatttatataggtaatcgctttcataaaatatattacccttaggaaatcaaatgattataagtcatatatgaaaacgttatatataattgatatgtttacatttgtaacttagatttctctttgatttccacatatataaatcttaaggaggagttttc |
| **Tfdx** | ataaaaataagaagcctgcatttgcaggcttcttatttttat |
| **Pcpthl** | GACTTTGTTAAAAAAGTTTAATAAATATAATTTGAATAAATGGTATAAATAGACAGATATTTAGAATATTATAGAAATTTTAATAAAAGACTTCTATAATAAAGCTAAATTATCTGTCTTTTTTTTCGAAAAGAGAAAAAATAATAAAAAAGATTGTTTAAAATTTAACAAAAAATATTGAAAGCTAGTAAATTTATGGTTATAATTAAATCAGTTAGAAAAAATATAGATTTTTG |
| **TecT1** | CCAGGCATCAAATAAAACGAAAGGCTCAGTCGAAAGACTGGGCCTTTCGTTTTATCTGTTGTTTGTCGGTGAACGCTCTC |
| **Pcpfdx** | aattaaatttttaatacggtataggggtattctttagcatgttaattctaattttactagaataggctaaatatgcttaaaagagatattaaatactttaaaaattagcaaaaatatgataatataaatatggta |
| **TbstyrS** | ATAATCAATCGTCCCTTCGTGTAAACGAAGGGGCGTTTTTTATTT |
| **chRNA** | gttttagagctagaaatagcaagttaaaataaggctagtccgttatcaacttgaaaaagtggcaccgagtcggtgcttttttt |

### S. pyogenes CRISPR array

Table S.4: Key parts of the mCRISPR array construct. DR=Direct repeat; tracrRNA= trans-activating CRISPR RNA.

| **Part** | **Sequence** |
| --- | --- |
| **DR** | GTTTTAGAGCTATGCTGTTTTGAATGGTCCCAAAAC |
| **tracrRNA** | GGAACCATTCAAAACAGCATAGCAAGTTAAAATAAGGCTAGTCCGTTATCAACTTGAAAAAGTGGCACCGAGTCGGTGCTTTTTTT |

### tRNA array

Table S.5: Key parts of the mtRNA array construct. Capitalized letters represent the sequence of the final tRNA obtained after post-transcriptional processing.

| **Name** | **Sequence** |
| --- | --- |
| **tRNA-Thr-TGT-1-1** | GCTGGCATAGCTCAATTGGTAGAGCAACTGACTTGTAATCAGTAGGTTATGGGTTCAATTCCTATTGCCAGCACCAattttggaggaattcccgagt |
| **tRNA-fMet-CAT-1-1** | CGCGGGGTGGAGCAGCTGGCAGCTCGTCGGGCTCATAACCCGAAGGTCGTAGGTTCAAGTCCTACCCCCGCAACCAgatttggcggaatagctcagct |

# Cas9 variants with alternative PAMs

Table S.6: Some of the published Cas9 variants and their associated PAMs.

| Cas9 variant | PAM | Reference |
| --- | --- | --- |
| VQR | NGA | (Kim et al., 2017; Kleinstiver et al., 2015) |
| EQR | NGAG | (Kim et al., 2017; Kleinstiver et al., 2015) |
| QQR1 | NAAG | (Anders et al., 2016) |
| iSpymac | NAA | (Chatterjee et al., 2020; Liu, Shan, et al., 2019) |
| xCas9 | NG/NGD | (Hu et al., 2018; Liu, Chen, et al., 2019) |
| Cas9-NG | NG | (Nishimasu et al., 2018) |
| SpG | NG | (Walton et al., 2020) |
| SpRY | N/NH | (Walton et al., 2020) |
